# RHYTHM-CENTRED MUSIC INTERVENTIONS FOR MOTOR COORDINATION AND REHABILITATION: A SCOPING REVIEW OF MECHANISMS, DESIGN PRINCIPLES, AND TECHNOLOGY-ASSISTED ASSESSMENT

**DOI:** 10.2340/jrm.v58.46015

**Published:** 2026-10-12

**Authors:** Yongfan SONG, Yaming WEI

**Affiliations:** 1 Institute of Physical Education Teaching and Research, Fuzhou University, Fuzhou, Fujian; 2 Research Center for Arts and Health, Xiamen Humanity Hospital, Xiamen, Fujian, China

**Keywords:** rhythm-centred music intervention, rhythmic auditory cueing, movement quality, motor rehabilitation, technology-assisted assessment, sensorimotor integration

## Abstract

**Objective:**

To map the evidence on rhythm-centred music interventions and movement-directed music-based strategies for motor coordination and rehabilitation, and to summarize their mechanisms, design principles, and technology-assisted assessment approaches.

**Methods:**

This scoping review followed the Joanna Briggs Institute framework and was reported in accordance with PRISMA-ScR. PubMed, Scopus, Web of Science, and PsycINFO were searched for English-language empirical studies published from January 2013 to December 2024. Studies were eligible if music or rhythmically structured auditory input was used as a movement-directed intervention and at least one motor, coordination, functional, or movement-quality outcome was reported.

**Results:**

Sixty-three studies were included. Coordination-related tasks were the most common targets, while lower-limb/gait function and fine-motor skills formed the largest movement categories. Rhythm was the most frequently used musical element, whereas melody, pitch, motivational music, active music making, and interactive digital systems served more context-dependent functions. Technology-assisted assessment, including sensors, motion capture, gait-analysis platforms, and music-interactive software, was the most common measurement approach.

**Conclusion:**

Rhythm-centred and music-based motor interventions are heterogeneous and should be interpreted as descriptive evidence patterns rather than definitive estimates of therapeutic efficacy. Future studies should improve terminology, intervention parameterization, population-specific interpretation, methodological transparency, and multimodal mechanistic validation.

## LAY ABSTRACT

Music is often used to support movement during training and rehabilitation, especially when rhythm helps people time and coordinate their actions. This scoping review summarized 63 studies published between 2013 and 2024 on music-based interventions for motor coordination and motor rehabilitation. Most studies focused on walking, lower-limb movement, or fine-motor tasks. Rhythm was the most common music element, and many studies used sensors, motion-capture tools, gait systems, or other technology to measure outcomes. The findings suggest that music may support movement through timing, sensorimotor coupling, attention, motivation, and feedback. More research is needed to test these approaches in specific patient groups and to improve individualized music-based rehabilitation design.

In motor training and rehabilitation, music can function through both temporal cueing and broader motivational or affective pathways. Rhythmically structured auditory input provides beat, tempo, and temporal regularity that can support movement timing, whereas complete music may also influence attention, arousal, and engagement. This distinction is important because a rhythm cue used to regulate gait, a motivational song used during exercise, and an interactive music-feedback system do not necessarily operate through the same mechanism. Compared with non-musical auditory signals, music can combine temporal predictability with motivational salience, making it a potentially useful but heterogeneous intervention medium for gait training, upper-limb movement, fine-motor tasks, and coordination-related activities (1–3).

Among musical components, rhythm is most directly linked to temporal aspects of motor control. Rhythmic auditory cues can provide an external timing scaffold that helps individuals initiate actions, maintain cadence, and adjust movement timing, thereby supporting rhythmic synchronization and sensorimotor coupling (1). Nevertheless, rhythm-based cueing should be distinguished from music-supported therapy, instrument playing, singing, sonification, and passive or motivational listening. These approaches may share musical material but differ in task demands, feedback structure, cognitive load, emotional engagement, and the extent to which movement is explicitly synchronized to an external beat.

The motor-facilitating effects of music can be understood from multiple interrelated theoretical and mechanistic perspectives. First, the theory of rhythmic synchronization proposes that external auditory beats can couple with an individual’s internal motor rhythms, thereby improving synchronization and temporal control during repetitive movements (1, 4). Second, theories of motor control indicate that auditory input plays an important role in movement planning, execution, and correction; related studies have also shown that auditory stimulation can activate brain networks associated with motor preparation and movement execution (1, 4, 5). In addition, from a broader psychobehavioural perspective, music may indirectly enhance training engagement and persistence by increasing attentional level, training motivation, and emotional arousal (6, 7). Overall, music, especially rhythm-centred musical stimulation, may jointly promote motor performance and functional recovery through multiple mechanisms, including temporal synchronization, sensorimotor integration, and psychobehavioural support.

Relevant research spans multiple domains, including neurorehabilitation, functional training in older adults, motor skill learning, and experimental motor control. The target populations also vary substantially, including healthy adults and individuals with stroke, Parkinson’s disease, cerebral palsy, developmental coordination disorder, multiple sclerosis, or other motor impairments (2). Intervention formats are similarly diverse, ranging from beat-based rhythmic auditory cueing and pre-recorded music listening to instrument-based participation, singing, sonification, interactive digital music systems, and hybrid feedback interventions (1). This heterogeneity makes it necessary to map not only whether music is used, but also which musical element is operationally central, whether movement is synchronized to sound, and how outcomes are measured.

Against this backdrop, a scoping review represented an appropriate methodological approach. Compared with systematic reviews or meta-analyses, which place greater emphasis on integrating effect sizes, scoping reviews are better suited to fields characterized by complex research populations, diverse forms of evidence, and substantial methodological heterogeneity (8). In the case of music-facilitated motor coordination and motor rehabilitation, the relevant literature extends across disciplines and application settings, encompassing not only clinical studies but also experimental investigations, technology development, and feasibility research. Accordingly, the field first requires a systematic mapping and structured synthesis of the available evidence. A scoping review can more clearly identify the motor domains that have received the greatest attention, the musical elements and selection rationales that have been adopted, how interventions have been delivered, and the indicators most commonly used to evaluate outcomes, thereby laying the groundwork for subsequent focused systematic reviews, mechanistic studies, and translational clinical research. The conceptual framework guiding this review is shown in Fig. 1.

**Fig. 1:**
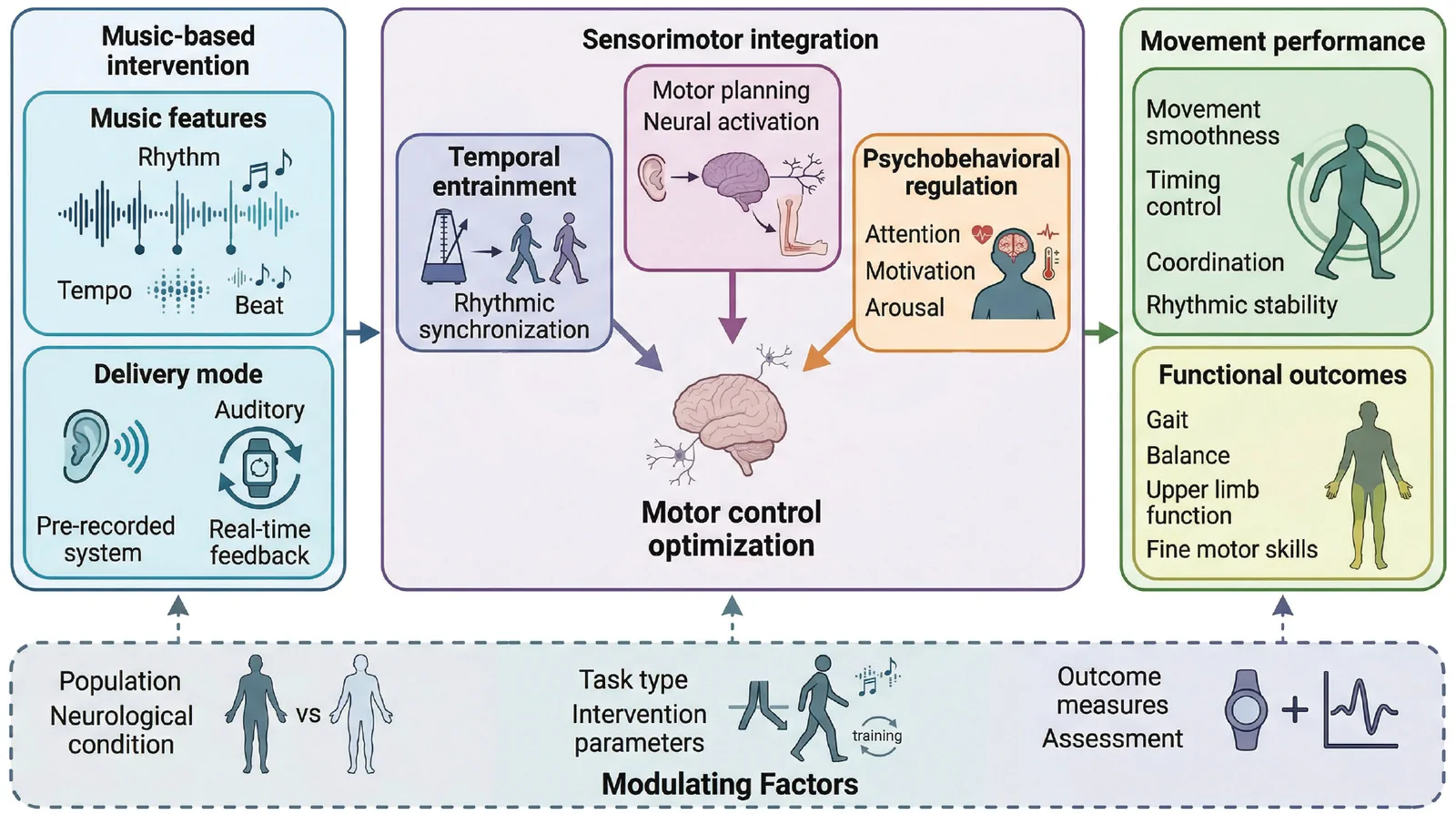
Conceptual framework of music-facilitated motor performance.

On this basis, the present review aimed to map the application characteristics, intervention taxonomy, key musical elements, delivery modes, and assessment methods of rhythm-centred and movement-directed music interventions in motor coordination and motor rehabilitation. Specifically, this study addressed 4 questions. First, which populations and motor domains have been examined in studies using music or rhythmically structured auditory input to support movement? Second, how can the included interventions be operationally classified, including rhythmic cueing, music listening, active music making, singing, sonification, and interactive digital systems? Third, which outcome measures and measurement tools have been used to evaluate movement-related effects? Fourth, what descriptive evidence patterns, methodological limitations, and future research priorities can be identified without overinterpreting the available heterogeneous evidence?

## METHODOLOGY

### Review design and reporting framework

This study adopted a scoping review approach to systematically examine the scope of application, principal characteristics, and methodological distribution of research on music intervention in motor coordination and motor function rehabilitation. The main reason for selecting a scoping review was the substantial heterogeneity in the existing literature regarding study populations, intervention formats, motor task types, and outcome measures (8). In addition, the relevant research contexts spanned multiple application settings, including rehabilitation training, functional motor tasks, and motor performance enhancement. Therefore, compared with systematic reviews or meta-analyses that focus on effect-size synthesis, a scoping review was better suited to mapping the evidence base, identifying research and intervention patterns, and revealing gaps in the current literature (8, 9).

The methodological procedures of this review followed the Joanna Briggs Institute guidance for scoping reviews and were reported in accordance with the Preferred Reporting Items for Systematic Reviews and Meta-Analyses extension for Scoping Reviews (PRISMA-ScR) (8, 10). These frameworks were adopted to enhance the transparency and methodological rigour of the review process and to ensure that the procedures for literature searching, screening, data extraction, and evidence synthesis were clearly defined and traceable.

Given substantial variation among the included studies in study design, intervention mode, and outcome measures, this study primarily used descriptive synthesis and categorical summarization rather than quantitative pooling. In other words, the focus of this review was not to estimate the overall effect size of music intervention, but to systematically summarize its main areas of application, core musical elements, modes of implementation, and commonly used outcome measures and measurement methods. Accordingly, no meta-analysis was conducted, and the findings were presented primarily as a narrative synthesis, supplemented by tables and figures.

### Population–Concept–Context (PCC) framework and inclusion and exclusion criteria

To improve the clarity of scope definition and strengthen the consistency of the literature screening process, this review used the PCC framework to establish the inclusion and exclusion criteria (8). The PCC framework is well suited to scoping reviews for the structured definition of study populations, core concepts, and application contexts, and is particularly useful for maintaining systematic and operational screening criteria when the research topic is broad and the evidence base is diverse (9).

For the Population component, this review included studies involving human participants, including both healthy populations and individuals with motor limitations or rehabilitation needs. No age restrictions were applied. Studies were eligible for inclusion if they involved motor performance, motor coordination, motor control, gait function, upper-limb activity, or fine-motor tasks. This review excluded animal studies and other research involving nonhuman subjects, as well as studies that did not involve any motor-related activity or motor function task.

For the Concept component, this review focused on music-related or rhythmically structured auditory stimulation used as a primary movement-directed intervention component. Eligible interventions were operationally classified into 5 non-mutually exclusive categories: (*i*) rhythmic auditory cueing or beat-based auditory stimulation designed to synchronize or regulate movement timing; (*ii*) complete music listening used during movement execution, gait training, exercise, or rehabilitation tasks; (*iii*) active music making, including instrument playing or singing, when movement practice was integral to the intervention; (*iv*) sonification, auditory feedback, or interactive digital music systems in which movement produced or modified sound; and (*v*) hybrid interventions combining music, rhythm, feedback, or conventional rehabilitation. Studies using music only as background ambience, relaxation, meditation, or emotion regulation were excluded unless the music directly served movement execution, motor training, functional recovery, or sensorimotor synchronization.

For the Context component, this review included studies conducted in the contexts of motor training, rehabilitation training, gait training, balance and coordination tasks, upper-limb function training, fine-motor training, and other functional movement activities. In other words, studies were eligible if their context was explicitly directed toward improving motor performance, enhancing motor coordination, or supporting motor rehabilitation. At the same time, included studies had to report at least one outcome measure related to movement quality, motor coordination, motor control, or other relevant functional outcomes; studies lacking such outcome measures were excluded.

Regarding study type and source restrictions, this review included empirical studies published in English, including quantitative, qualitative, and mixed-methods research. Eligible studies had to report identifiable methodological information and outcome measures to support subsequent data extraction and categorical synthesis. Excluded publications comprised reviews, commentaries, theoretical articles, conference abstracts, editorials, and articles lacking sufficient methodological detail or inaccessible full study information. For duplicate publications, only the version containing the most complete information and best meeting the inclusion criteria was retained.

Based on the PCC framework described earlier, this review focused on the evidence regarding the application of music intervention in motor coordination and motor function rehabilitation and used uniform inclusion and exclusion criteria to improve the specificity and consistency of the screening process. The corresponding screening criteria are presented in Table I.

**Table I:** Eligibility criteria based on the PCC framework

PCC domain	inclusion criteria	Exclusion criteria
Population	Human participants, including healthy individuals and people with motor impairment or rehabilitation needs	Animal/non-human studies; studies without movement-related tasks or motor-function outcomes
Concept	Primary music-related or rhythmically structured auditory interventions, including full music, rhythmic/melodic stimuli, or structured cues for movement	No music/auditory intervention; music used only as background, relaxation, meditation, emotion regulation, or a non-core component
Context	Motor training, rehabilitation, gait, balance/coordination, upper-limb, fine-motor, or other functional movement contexts with movement-related outcomes	No outcomes related to movement quality, motor control, coordination, or functional performance
Study types and sources	Peer-reviewed English empirical studies, including quantitative, qualitative, and mixed-method designs	Reviews, commentaries, editorials, conference abstracts, theoretical papers, duplicate publications, or studies with insufficient methodological detail

Note: PCC = Population, Concept, and Context; eligible study types included quantitative, qualitative, and mixed-method studies.

### Information sources and search strategy

This study systematically searched multiple internationally recognized databases to identify, as comprehensively as possible, relevant studies on music interventions in motor coordination and motor function rehabilitation. The databases searched were PubMed, Scopus, Web of Science, and PsycINFO. These databases cover medicine, sports science, psychology, and interdisciplinary research, thereby enhancing the breadth and representativeness of the search.

The search period was defined as January 2013 to December 2024, and all database searches were completed in January 2025. The 2013 starting year was selected because the previous decade marked a clear increase in digital motor assessment, wearable sensing, music-interactive rehabilitation systems, and contemporary rhythmic auditory cueing research, making it an appropriate window for mapping current intervention design and assessment pathways. The end date was aligned with the included-study distribution and the PRISMA flow diagram.

The search strategy was constructed around 3 core conceptual modules and adapted to each database: music/rhythm terms (music, rhythm, rhythmic auditory stimulation, rhythmic auditory cueing, melody, auditory stimulation), motor terms (motor coordination, motor control, movement quality, gait, balance, upper limb, fine motor, movement performance), and rehabilitation/training terms (rehabilitation, motor training, exercise, functional outcome, task performance). A representative Boolean structure was: (music OR rhythm* OR rhythmic auditory stimulation OR rhythmic auditory cueing OR melody OR auditory stimulation) AND (motor coordination OR motor control OR movement quality OR gait OR balance OR upper limb OR fine motor OR movement performance) AND (rehabilitation OR motor training OR exercise OR functional outcome OR task performance). Detailed information on the technical tools used in the included studies is provided in Table SI. Database-specific adaptations for PubMed, Scopus, Web of Science, and PsycINFO are provided in Table SII, and the PRISMA-ScR checklis.

In addition to the database search, backward reference searching of included articles was used to check potentially relevant studies. Supplementary manual searching was used as a verification step for highly relevant articles and did not yield additional eligible records beyond those shown in the PRISMA flow diagram. Before formal screening, all search results were imported into reference management software, duplicates were removed, and the remaining records proceeded to title and abstract screening.

### Study selection

Study selection was conducted in stages according to the predefined inclusion and exclusion criteria. First, all records retrieved from the databases were compiled, and duplicates were removed after import into the reference management software. After deduplication, title and abstract screening were conducted to exclude studies that were clearly irrelevant to the review topic. Full texts were then retrieved and assessed for the studies retained after the initial screening to further determine whether they met the inclusion criteria of this review.

The study selection process was completed independently by 2 reviewers. Both reviewers screened titles/abstracts and full texts according to the predefined PCC criteria and intervention taxonomy. Disagreements were discussed until consensus was reached; when consensus could not be achieved, a third reviewer was consulted. Because the archived screening file retained consensus decisions rather than the complete pre-consensus coding matrix, a reliable retrospective Cohen’s kappa could not be calculated; this limitation is now explicitly acknowledged in the Discussion. The number of records at each screening stage and the full-text exclusion reasons are summarized in Fig. 2 and Table SIII.

**Fig. 2:**
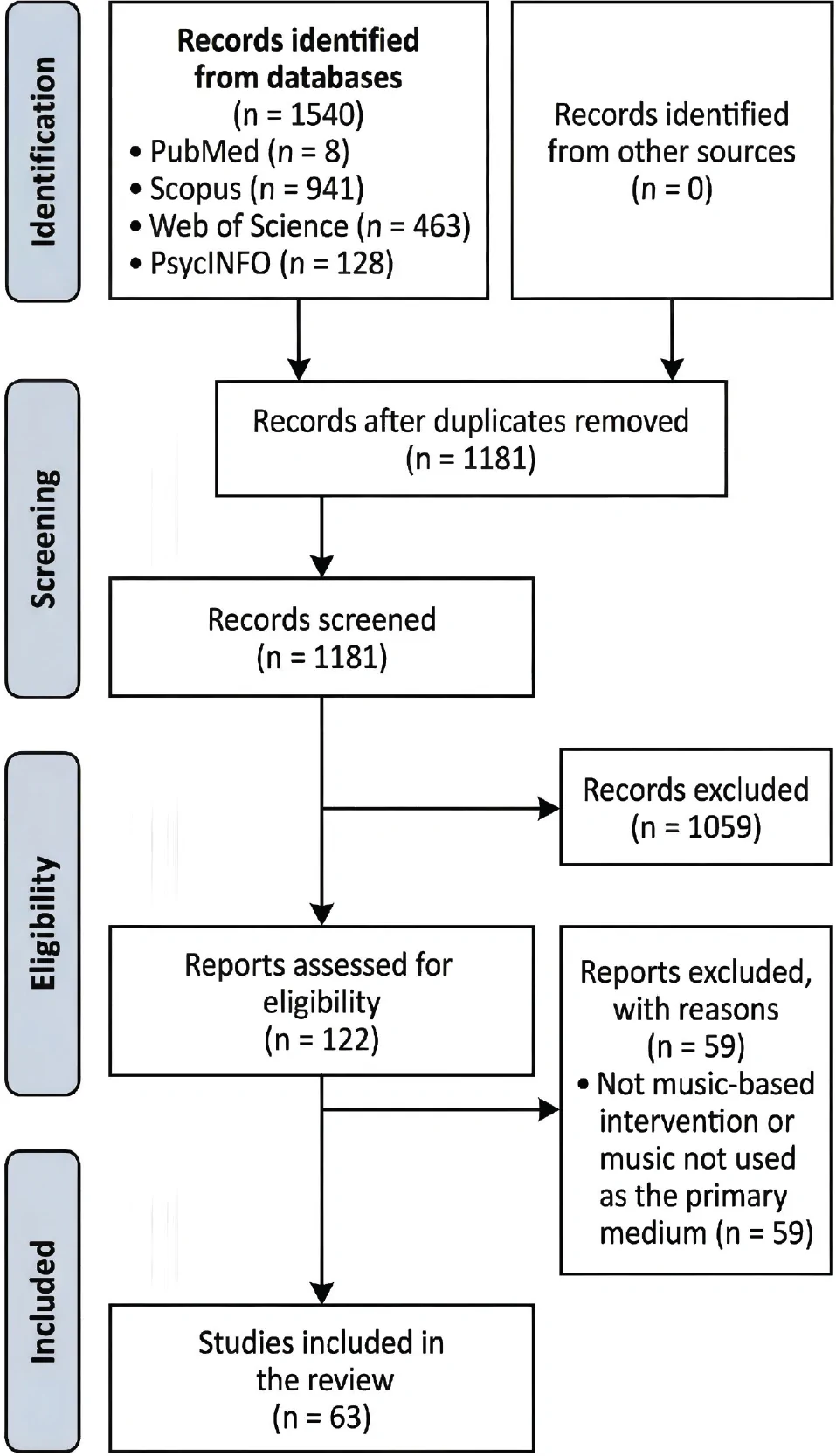
PRISMA 2020 flow diagram of the study selection process, including records identified through database searching and supplementary source checking.

The entire selection process was presented using a PRISMA 2020 flow diagram (see Fig. 2). A total of 1,540 records were identified from databases, including PubMed (*n* = 8), Scopus (*n* = 941), Web of Science (*n* = 463), and PsycINFO (*n* = 128). After duplicate removal, 1,181 records were screened, 1,059 records were excluded at title/abstract screening, and 122 reports were assessed for eligibility. Fifty-nine full-text reports were excluded because the intervention was not music-based or music was not used as the primary movement-directed medium. Finally, 63 studies were included in the review.

### Data extraction and variable organization

To systematically organize the information from the included studies, this review used a standardized data charting form to extract and synthesize the relevant data (8). The data charting form was used to consistently record the basic characteristics of each study, intervention design, target movement types, outcome measures, and main findings, thereby ensuring consistent and comparable data organization across studies.

The extracted information mainly included the following aspects. First, basic study characteristics, including the authors, year of publication, country or region in which the study was conducted, and study design. Second, sample characteristics, including participants’ age, sex, health status, and population type. Third, intervention characteristics, including the type of target movements, the musical elements used, the mode of music delivery, intervention duration, intervention frequency, and the way music was integrated into the activity. Fourth, outcome and assessment information, including the primary outcome measures, movement- or function-related assessment tools, technology-assisted measurement approaches, and other objective or subjective measurement methods. Fifth, study results, including the main findings and related conclusions reported in the articles.

Before formal data extraction, the research team first selected a subset of studies for pilot extraction to assess the applicability of the data charting form and the clarity of the classification framework. Based on the issues identified during this pilot process, the variable names, classification hierarchy, and recording procedures were appropriately revised to improve the consistency and completeness of the subsequent formal extraction. After these revisions were completed, all included studies were systematically extracted and organized.

To facilitate subsequent analysis, after data extraction was completed, this study further classified the extracted information by study characteristics, target movement domains, music intervention design, and assessment methods, thereby providing a foundation for the subsequent descriptive summary and categorical synthesis (11).

Before synthesis, a coding framework was finalized to improve consistency across studies. Target-movement categories were assigned according to the primary movement trained or assessed in each study. Lower-limb/gait, upper-limb, fine-motor, and body-coordination categories were treated as non-mutually exclusive when a study involved more than 1 movement domain. Musical-element coding prioritized the element explicitly used to guide, cue, structure, or provide feedback for movement. The term “sensorimotor responsiveness” was used to describe agility, response speed, or sensory-motor reactivity when these constructs were reported in the original studies.

### Data synthesis and analysis

This review integrated and analysed the included studies by combining descriptive statistics with categorical synthesis. First, the basic characteristics of the studies were summarized descriptively, including the number of studies, publication year, geographic distribution, study design, and sample characteristics, to present the overall distribution and developmental trends of research in this field.

On this basis, the included studies were categorized and synthesized in relation to the review questions, with the analysis focusing on the following aspects: target movement domains, musical elements, modes of music delivery, intervention design characteristics, and major assessment tools. Specifically, the domains of target movements included lower-limb, upper-limb, whole-body coordination, and fine-motor tasks. The characteristics of the music intervention included core musical elements such as rhythm and melody, as well as delivery modes including listening-based, performance-based, and hybrid formats. Assessment methods covered technology-assisted assessment, standardized objective measurements, neurophysiological indicators, and subjective evaluations. Through this classification, the study sought to identify the principal patterns and shared characteristics in the existing literature.

In addition to the overall descriptive summary and categorical synthesis, this study also conducted basic cross-comparative analyses to examine differences in intervention characteristics across research contexts and movement types. These analyses focused on 2 sets of comparisons. The first concerned differences between rehabilitation contexts and training/motor performance contexts in terms of music design, delivery modes, and assessment methods. The second concerned differences between lower-limb/gait tasks and upper-limb/fine-motor tasks in terms of core musical elements and measurement approaches. This part of the analysis was intended to reveal the distributional characteristics of music intervention strategies and assessment pathways across different application scenarios, without involving formal statistical inference.

Given the substantial heterogeneity among the included studies in terms of study populations, intervention modes, outcome measures, and measurement tools, no formal effect-size pooling or meta-analysis was conducted. Accordingly, the findings were presented primarily as a narrative synthesis, supplemented by tables and figures, to summarize the application patterns, evidence structure, and research gaps in music interventions for motor coordination and motor function rehabilitation.

Consistent with the mapping purpose of a scoping review, no formal risk-of-bias score was used to exclude or weight studies. Study design was nevertheless charted and considered during interpretation: randomized and crossover trials were treated as stronger evidence for intervention effects than single-group, case, feasibility, or exploratory technology-development studies. These differences are considered in the Discussion to avoid presenting descriptive patterns as established therapeutic efficacy.

## Results

### General characteristics of the included studies

After the systematic search and screening process, a total of 63 studies were ultimately included in this review (see Appendix S1 for details). These studies constituted the evidence base for music intervention in motor coordination and motor function rehabilitation, encompassing diverse regions, publication periods, and study designs. Overall, the included studies showed several notable patterns in terms of geographic distribution, publication year, and study design.

In terms of geographic distribution, the included studies were concentrated primarily in Europe and North America. Europe accounted for the largest number of studies, with 35, followed by North America with 18, Asia contributed 8 studies, and Oceania contributed 2. Overall, the current body of research was distributed primarily across Europe and North America, with relatively few studies conducted in other regions.

Regarding publication year, the number of included studies showed an overall upward trend. When grouped by period, 9 studies were published between 2013 and 2014; the number increased to 26 between 2015 and 2019, and further to 28 between 2020 and 2024. These findings indicate that, over the past decade, the application of music intervention in movement-related research has continued to attract attention, with the number of relevant studies gradually increasing.

Regarding study design, the included literature consisted primarily of interventional studies. Among these, randomized controlled trials and single-group pre-test–post-test studies were the most common designs, with 15 studies each, followed by 10 single-group post-test studies. In addition, there were 7 experimental studies, 7 mixed-methods studies, and 4 cross-sectional surveys. The remaining studies included 3 quantitative studies, 1 case series, and 1 randomized crossover trial. Overall, the current evidence has been derived mainly from studies that focus on intervention implementation and outcome observation.

In summary, a considerable body of evidence has accumulated on music interventions in the fields of motor coordination and motor function rehabilitation; however, the existing research has remained largely concentrated in Europe and North America, and interventional designs have predominated. The general characteristics of the included studies are presented in Table II.

**Table II:** General characteristics of included studies

Characteristic	Category	Number of studies (*n*)
Country/region	Europe	35
	North America	18
	Asia	8
	Oceania	2
Publication year	2013–2014	9
	2015–2019	26
	2020–2024	28
Study design	Randomized controlled trial	15
	One-group pre–post study	15
	One-group post-only study	10
	Experimental study	7
	Mixed-methods study	7
	Cross-sectional survey	4
	Quantitative study	3
	Case series	1
	Randomized cross-over trial	1

RCT: randomized controlled trial. Other designs include case series, randomized crossover trials, and other small-sample study designs.

### Study populations and domains of target movements

The domains of target movements addressed in the included studies showed a clear concentration in coordination-related outcomes (Fig. 3A). Coordination was the most frequently targeted ability, appearing 55 times, followed by speed and sensorimotor responsiveness with 12 occurrences each, whereas strength and flexibility were addressed less often, appearing only 4 and 2 times, respectively. Sensorimotor responsiveness was used to describe outcomes such as agility, response speed, or sensory-motor reactivity when these were the primary movement-related constructs in the original studies.

**Fig. 3:**
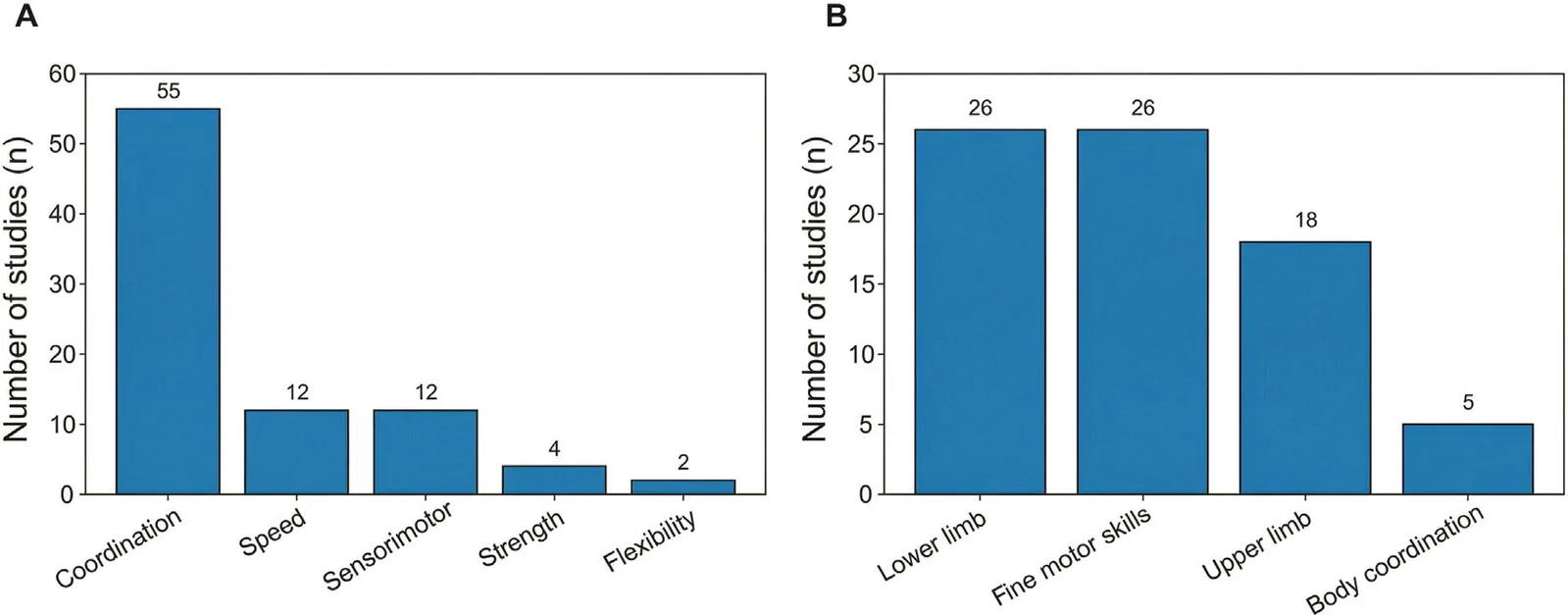
Distribution of target movement capacities and movement types in music intervention. (A) Distribution of movement capacity dimensions addressed in the included studies; (B) Distribution of body regions and functional movement types.

Further analysis by body region and functional category showed that the included studies were concentrated mainly in 2 areas: lower-limb movements and fine-motor skills, with 26 studies in each category (Fig. 3B). These were followed by upper-limb movements, which were examined in 18 studies, whereas body coordination movements were addressed least frequently, with only 5 studies. This pattern indicates that the strongest body of evidence has focused on gait, lower-limb activity, and hand- or finger-related fine-motor tasks, whereas studies addressing whole-body coordination or more complex patterns of bodily coordination have remained comparatively limited.

This distribution closely aligns with the application settings for music intervention. Studies of lower-limb movements were often associated with gait training, rhythmic walking, and motor rehabilitation, whereas studies of fine-motor skills were more commonly associated with upper-limb functional recovery, hand training, and music-interactive tasks. By comparison, whole-body coordination tasks typically involve more complex movement organization and greater demands on measurement, which may explain their smaller representation in the current literature. Overall, the application of music intervention across domains of target movements has shown a relatively stable orientation, with coordination control as the central focus and a primary emphasis on lower-limb and fine-motor tasks (Table III).

**Table III:** Targeted motor domains and functional movement categories in music-based interventions

Category	Subcategory	Number of studies (*n*)
Motor performance domains	Coordination	55
	Speed	12
	Sensorimotor responsiveness*	12
	Strength	4
	Flexibility	2
Movement location/function	Lower limb	26
	Fine motor skills	26
	Upper limb	18
	Body coordination	5

Some studies addressed more than 1 motor domain or movement category; therefore, the total counts may exceed the number of included studies (*n* = 63).

* Sensorimotor responsiveness refers to agility, response speed, or sensory-motor reactivity depending on the original study context.

### Musical elements and intervention design

From the perspective of music selection logic, the included studies showed a clear tendency towards function-oriented decision-making, whereas participants’ subjective preferences were rarely used as the primary basis for selection (Fig. 4A). Among the included studies, the most common basis for music selection was rhythmic characteristics, reported in 46 studies (73%). These studies typically selected music based on tempo, beat regularity, or rhythmic complexity to match the requirements of the target movement task. The second most common approach was interactive feedback-based music selection, reported in 14 studies (22%), in which the musical content was dynamically adjusted according to participants’ performance during training or their therapeutic progress. By contrast, only 2 studies (3%) selected music based on structural musical elements, and only 1 study (2%) selected music based on participants’ preferences or cultural background. Overall, the current literature has relied primarily on function-driven strategies for music selection.

**Fig. 4:**
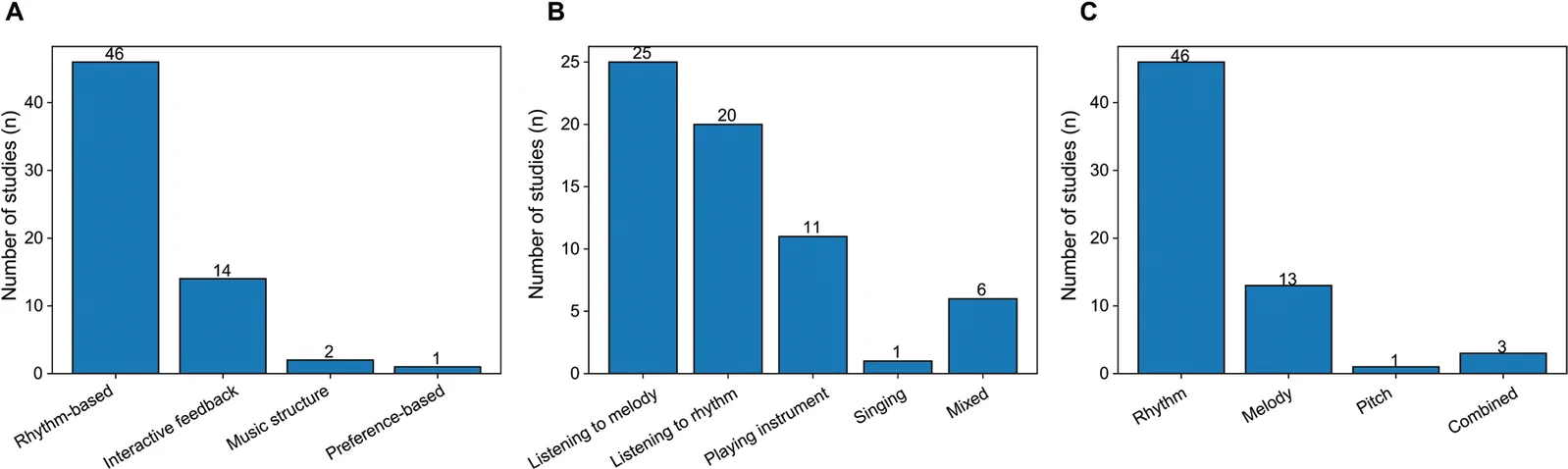
Distribution of musical elements and intervention design characteristics. (A) Distribution of music selection logic; (B) Distribution of music delivery modes; (C) Distribution of core musical elements.

In terms of delivery mode, the existing interventions mainly fell into 3 categories: listening-based interventions, active music making, and hybrid interventions (Fig. 4B). The most common format was melody listening, which appeared in 25 studies (40%), followed by rhythm listening, reported in 20 studies (32%). In addition, 11 studies (17%) involved instrument playing, only 1 study (2%) involved singing, and 6 studies (9%) used hybrid interventions. Overall, listening-based interventions and rhythmic cueing approaches constituted the most common implementation modes, whereas interventions involving active performance accounted for a relatively smaller proportion.

Regarding core musical elements, rhythm was the most frequently used component for facilitating movement (Fig. 4C). Rhythm was identified as the primary musical element in 46 studies (73%), representing the highest proportion among all musical elements. This was followed by melody, which was reported in 13 studies (20%). Cases in which pitch alone served as the key element were extremely rare, with only 1 study (2%). In addition, 3 studies (5%) adopted an integrated strategy, simultaneously combining multiple musical elements to support motor tasks. Taken together, the existing literature has treated rhythm as the central musical element, with melody more often serving a supportive role, whereas fully integrated musical strategies have remained relatively uncommon.

### Outcome measures and assessment methods

The outcome measures examined in the included studies primarily focused on improvements in motor and functional performance, including coordination, gait, upper-limb function, fine-motor ability, and task-related performance. Different studies selected corresponding outcomes according to the target movement tasks and application contexts. For example, gait studies typically focused on step length, gait speed, and gait symmetry, whereas studies of upper-limb and fine-motor function more often assessed hand dexterity, movement accuracy, reaction time, and task completion performance. Overall, the current literature has evaluated the effects of music intervention primarily along 2 dimensions: motor control quality and performance on functional tasks.

In terms of assessment approaches, the included studies could be broadly classified into 4 categories: technology-assisted assessments, objective standardized measures, neurophysiological indicators, and subjective assessments. Among these, technology-assisted assessment was the most common measurement approach, appearing 52 times in the included studies, markedly more often than the other categories. Objective standardized measures appeared 18 times, neurophysiological indicators 6 times, and subjective assessments were the least common, with only 2 occurrences. This distribution indicates that research on music-facilitated movement has clearly shifted towards the use of objective, digital, and high-resolution measurement tools to capture changes in motor performance (Table IV).

**Table IV:** Outcome domains and assessment methods used in the included studies

Assessment category	Description	Representative indicators/tools	Frequency (*n*)
Technology-assisted assessments	Objective digital or instrumented assessment of motor performance	Inertial sensors; motion capture; gait analysis; music-interactive software; tablet/touch devices; audio/tapping tools	52
Objective standardized measures	Standardized clinical scales or functional tests for motor and functional performance	Nine-Hole Peg Test, Box and Block Test, Fugl-Meyer Assessment, Timed Up and Go Test, Berg Balance Scale	18
Neurophysiological indicators	Neural or physiological measures of music-related motor mechanisms	EEG, MEG, ECG, EMG, event-related potentials	6
Subjective assessments	Observer/researcher ratings of movement synchrony or functional improvement	Ratings of rhythm-movement synchrony or subjective motor improvement	2

Note: Some studies used more than 1 type of assessment; therefore, the total frequencies may exceed the number of included studies (*n* = 63).

Regarding technology-assisted assessment, the relevant studies used a wide range of digital and instrument-based tools. Common devices included inertial sensors and wearable sensing systems, which were used to record joint angles, movement speed, and gait cycles; motion capture systems, which were used to track gait, limb kinematics, and motor coordination; gait analysis systems, which were used to assess step length, cadence, walking speed, and gait symmetry; and music-interactive software and digital platforms, which were used to record keystroke accuracy, rhythmic synchronization, reaction time, and performance on music-related tasks. In addition, tablet devices, touch-based interfaces, and audio-tapping synchronization recording tools were also used to measure fine-motor and rhythmic tasks. Overall, sensors, motion capture systems, gait analysis systems, and music-interactive software were the most commonly used tools for technology-assisted assessment in this field.

In addition to technology-assisted assessment, standardized clinical scales and objective functional tests play an important role in this field. Commonly used tools included the Nine-Hole Peg Test, Box and Block Test, Fugl-Meyer Assessment, Timed Up and Go Test, and Berg Balance Scale, all of which enabled structured evaluation of intervention effects across dimensions such as hand function, upper-limb activity, walking ability, and balance control. By contrast, neurophysiological indicators were used less frequently and primarily included electroencephalography, magnetoencephalography, electrocardiography, electromyography, and event-related potentials. Subjective assessments accounted for the smallest proportion and mainly consisted of observational ratings of rhythm-movement synchronization or the degree of motor improvement. Overall, the current literature showed a clear tendency to rely primarily on technology-assisted assessment, supplemented by objective standardized measures, whereas neurophysiological indicators and subjective assessments remained relatively underrepresented. A summary of the outcome measures and assessment methods is presented in Table IV, and detailed information on the technical tools is provided in Table SI.

### Evidence patterns and cross-domain comparisons

A synthesis of the included studies revealed design differences across application contexts, but these differences should be interpreted as descriptive frequency patterns rather than formal subgroup effects. The added Table SIV summarizes the available frequencies and proportions for major design and assessment variables. Overall, rhythm was the primary musical element in 46 of 63 studies (73%), melody in 13 studies (20%), integrated musical strategies in 3 studies (5%), and pitch in 1 study (2%). Listening-based delivery predominated, including melody listening in 25 studies (40%) and rhythm listening or rhythmic cueing in 20 studies (32%), whereas instrument playing, singing, and hybrid interventions accounted for 11 (17%), 1 (2%), and 6 (9%) studies, respectively.

From the perspective of target movement type, lower-limb/gait studies and upper-limb/fine-motor studies showed different implementation pathways. Lower-limb studies (*n* = 26) were more commonly linked to rhythmic auditory cueing, cadence regulation, gait speed, step length, and gait symmetry, and they frequently used gait-analysis platforms, inertial sensors, or motion capture. Fine-motor studies (*n* = 26) and upper-limb studies (*n* = 18) more often involved instrument-based, tablet-based, or music-interactive tasks, with outcomes such as hand dexterity, movement accuracy, reaction time, and rhythmic synchronization. Body-coordination tasks were less represented (*n* = 5), indicating that complex whole-body coordination remains a smaller evidence area.

Overall, the current body of research indicates that rhythm-centred and movement-directed music interventions are task-dependent. Gait and rehabilitation studies most often pair rhythmic cueing with standardized functional or digital gait assessment, whereas upper-limb and fine-motor studies more often pair active or interactive musical engagement with task-performance and precision measures. These patterns support the need for intervention taxonomies, domain-specific parameter reporting, and cautious population-specific interpretation.

## DISCUSSION

### Main findings and overall characteristics of the evidence

This review systematically synthesized the research evidence on music interventions in motor coordination and motor function rehabilitation and identified several relatively consistent patterns across the literature.

First, rhythm was the most frequently reported musical element and appeared especially relevant to temporal synchronization and coordination tasks. Second, the literature was concentrated mainly on lower-limb/gait function and fine-motor skills, whereas whole-body coordination and several diagnosis-specific populations were represented by fewer studies. Third, technology-assisted assessment was common, reflecting the field’s movement toward sensors, motion capture, gait analysis, and digital interactive platforms. Fourth, intervention parameters, including tempo, rhythmic complexity, synchronization requirements, session duration, training frequency, and progression strategy, were inconsistently reported.

Overall, the current evidence suggests a developing research model centred on rhythm-oriented cueing, task-specific music engagement, and technology-assisted assessment. However, because evidence quality, population characteristics, intervention dose, and outcome measures varied markedly, these patterns should be interpreted as a map of current research activity rather than confirmation of uniform clinical effectiveness.

### Mechanistic interpretation of rhythm-dominant motor facilitation

Rhythm occupied a prominent position because it provides temporal predictability for movement execution. From an entrainment perspective, external beats can align with internal motor timing and support repetitive actions such as walking, stepping, or tapping (1, 2). From a predictive-coding or active-sensing perspective, regular auditory structure may reduce temporal uncertainty, helping the nervous system anticipate when an action should occur and update movement timing when auditory and motor signals diverge. These mechanisms are particularly relevant to gait and other cyclic motor tasks.

Motor-learning theory further suggests that rhythm may support practice by structuring repetitions, providing external knowledge of timing, and reducing attentional demands once movement becomes more automatic. In rehabilitation settings, these effects may interact with neuroplasticity by increasing task repetition, salience, feedback, and engagement. At the same time, the benefits of music are not purely rhythmic: motivational and affective components may improve adherence, whereas interactive musical feedback can make errors more perceptible and may support online motor correction.

Neurophysiological evidence remains important but still limited. Studies using EEG, MEG, ECG, EMG, event-related potentials, PET, or other neuroimaging approaches suggest that music-facilitated movement may involve auditory–motor coupling, cortical–subcortical timing networks, motor preparation, and arousal regulation. However, these modalities were used in a minority of included studies, and the evidence was too heterogeneous to specify a single neural pathway. Recent studies of auditory-motor entrainment after stroke and autonomous closed-loop rhythmic auditory stimulation in Parkinson’s disease further highlight the translational potential of rhythm-based digital systems (12–14), but they also underscore the need for careful diagnosis-specific interpretation.

Overall, rhythm should be viewed as 1 important mechanism within a broader multisystem model involving temporal prediction, sensorimotor coupling, motor learning, feedback, motivation, and technological adaptation. This interpretation avoids treating all music-based interventions as mechanistically identical.

### Application value across motor domains and populations

The findings of this review indicated that music intervention showed a certain degree of functional differentiation across motor domains. In gait and lower-limb training, rhythmic music demonstrated particularly strong application value. Rhythm-based auditory cueing can provide a stable temporal structure for gait, thereby improving cadence, step length, and gait symmetry (15, 16). This characteristic is especially important in neurorehabilitation contexts, in which it can serve as a complementary approach to conventional rehabilitation training to support improvements in gait stability and walking efficiency (15).

In upper-limb and fine-motor training, active musical engagement showed considerable potential for application (17). Through instrument playing or music-based interactive tasks, individuals can receive immediate feedback as they execute movements, thereby promoting hand dexterity, movement accuracy, and bilateral coordination. This type of intervention can transform repetitive training into tasks with explicit goals and structured feedback, thereby enhancing the specificity of training.

In addition, music intervention may also positively influence training engagement (7). Compared with conventional training approaches, music can increase the appeal and sustainability of activities, thereby supporting individuals’ motivation to participate during long-term training or rehabilitation. At the same time, with the development of digital technologies, the use of sensors, motion capture systems, and music-interactive software has enabled music intervention to be integrated with real-time feedback mechanisms, thereby improving, to some extent, its adaptability and implementation flexibility.

Overall, rhythm-driven interventions were more commonly used in gait and lower-limb tasks, whereas interactive musical engagement was more frequently applied in upper-limb and fine-motor training, reflecting meaningful differences in the application pathways of music interventions across motor domains.

Population-specific interpretation is also necessary. In stroke rehabilitation, auditory-motor entrainment and rhythmic cueing may be most relevant to gait symmetry, walking speed, and motor relearning, but outcomes may depend on lesion characteristics, time since stroke, and baseline walking ability. In Parkinson’s disease, rhythmic auditory stimulation is closely related to cadence regulation, freezing of gait, and closed-loop cueing, yet responsiveness may vary with disease stage, medication state, and cognitive load. In cerebral palsy, developmental coordination disorder, and multiple sclerosis, rhythm-based or interactive musical interventions may need different task progressions because the underlying motor-control limitations differ. Findings from healthy participants or performance-training contexts therefore should not be generalized directly to clinical rehabilitation without considering diagnosis, impairment mechanism, and intervention dose.

### An integrated mechanistic model of music-facilitated movement

Based on the findings of this review, an integrated Input–Process-–Output (IPO) model can be constructed to explain the pathways through which music facilitates motor performance across different contexts (Fig. 5).

**Fig. 5:**
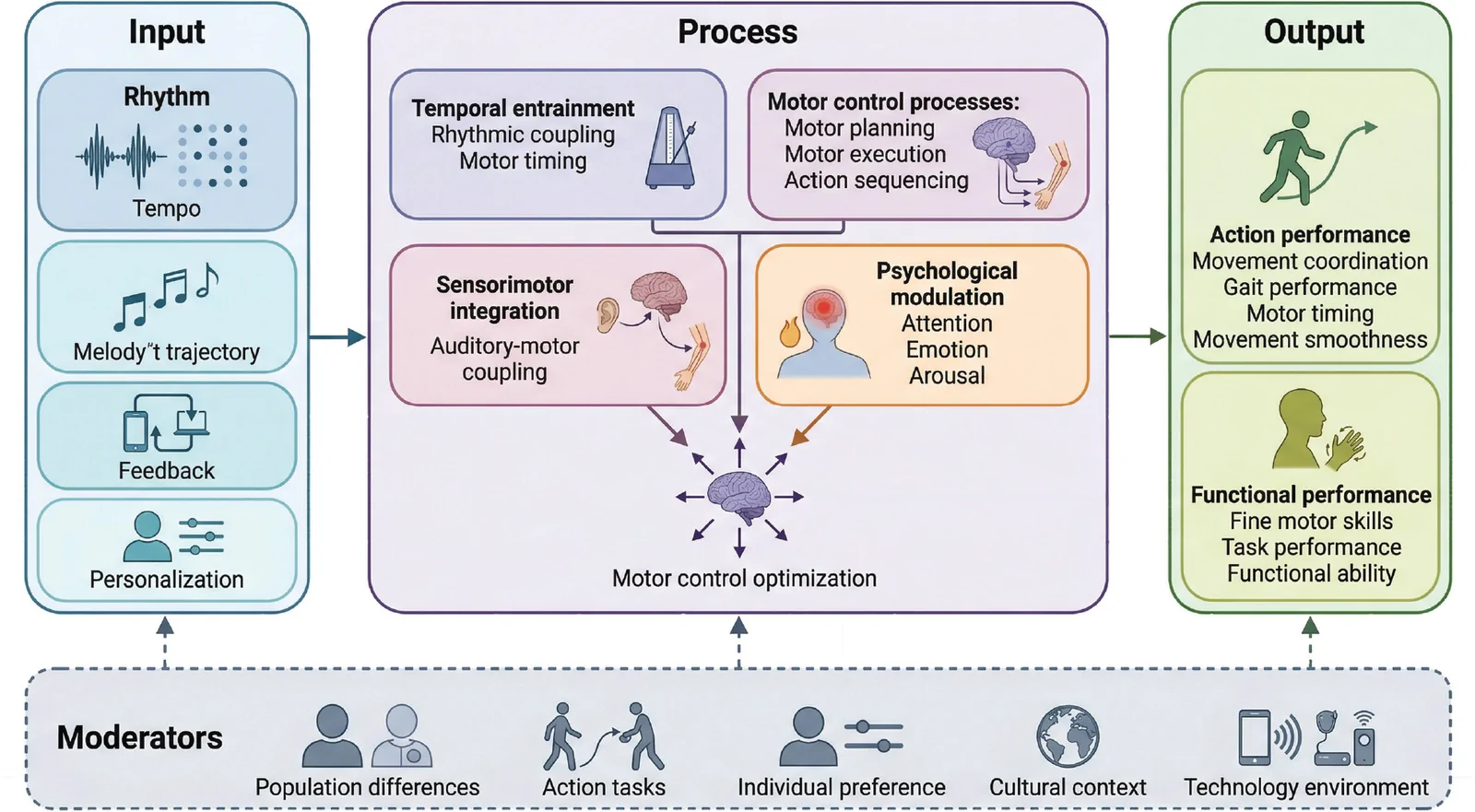
Schematic illustration of an integrated IPO model of music-facilitated motor improvement.

At the Input level, rhythm-centred music intervention consists of several key elements, including beat, tempo, rhythmic complexity, melody, feedback, and individualized preference. Among these, rhythm provides an external temporal reference; tempo influences movement pace; rhythmic complexity determines synchronization demands; melody and harmony may support engagement or sequential cueing; and feedback and individualized design enhance adaptability. Reporting these parameters is essential because interventions with the same label may impose very different motor and cognitive demands.

At the Process level, music influences motor performance through multiple mechanisms. Rhythmic synchronization (i.e., entrainment) is the core mechanism, establishing temporal coupling by aligning external beats with internal motor rhythms. At the same time, music also participates in the organization of motor planning and movement execution, making movement more fluid and predictable. In addition, music can indirectly affect movement efficiency by modulating attentional level and emotional state. At the neural level, music facilitates sensorimotor integration by strengthening the coupling between the auditory and motor systems, thereby enhancing motor control precision.

At the Moderator level, the effects of music intervention are shaped by factors such as population characteristics, task type, individual preferences, cultural background, and the technological environment. These factors determine the degree of fit between musical input and the operative processes, thereby influencing intervention outcomes. For example, rhythmic cueing tends to be particularly effective for gait tasks, whereas in fine-motor tasks it often needs to be combined with interactive feedback.

At the Output level, music intervention is ultimately reflected in improvements in coordination, gait performance, fine-motor ability, and task-related functional performance. This model indicates that music-facilitated movement arises from the joint action of multiple elements and mechanisms rather than from a single driving factor.

### Research limitations, evidence gaps, and future priorities

Although this review systematically mapped research on rhythm-centred and movement-directed music interventions, several limitations should be acknowledged. First, the included studies were heterogeneous in participant population, diagnosis, intervention format, dose, outcome measure, and study design, which limited the strength of cross-study comparison. Second, although 2 reviewers screened studies independently and resolved disagreements by consensus, the archived screening file did not retain a complete pre-consensus coding matrix; therefore, reliable retrospective kappa statistics could not be calculated. Third, no formal risk-of-bias tool was used to exclude or weight studies, and conclusions should therefore be interpreted as descriptive evidence mapping rather than graded evidence of efficacy. Fourth, this review included only English-language empirical studies, which may have introduced language and publication bias.

From the perspective of evidence structure, several gaps remain. Terminology and classification systems are not yet standardized, and intervention labels such as rhythmic auditory cueing, music-supported therapy, music listening, sonification, and interactive musical feedback are sometimes used inconsistently. Reporting of intervention parameters is also insufficient: many studies did not provide enough detail on tempo, beat salience, rhythmic complexity, synchronization requirements, session duration, training frequency, progression strategy, or individualization. In addition, population-specific evidence remains uneven, and neurophysiological studies are still too limited to fully explain the neural mechanisms through which music facilitates movement.

In light of these limitations and gaps, future research should advance in several directions. First, studies should use clearer intervention taxonomies and report rhythm/music parameters in sufficient detail for replication. Second, diagnosis-specific studies are needed to identify which strategies are appropriate for stroke, Parkinson’s disease, cerebral palsy, developmental coordination disorder, multiple sclerosis, healthy ageing, and performance-training contexts. Third, digital systems should be evaluated not only for technical precision but also for accessibility, implementation cost, clinician training requirements, home-based usability, data privacy, and long-term adherence. Fourth, future trials should integrate behavioural outcomes with neurophysiological and digital assessment to clarify mechanisms while avoiding overinterpretation of small or exploratory studies.

Overall, future research needs to move beyond asking whether music intervention is effective in general and instead determine which rhythm-centred or music-based strategy is appropriate for which population, task, dose, technological context, and mechanism. This shift would help the field develop toward more precise, transparent, and clinically translatable intervention design (Fig. 6).

**Fig. 6:**
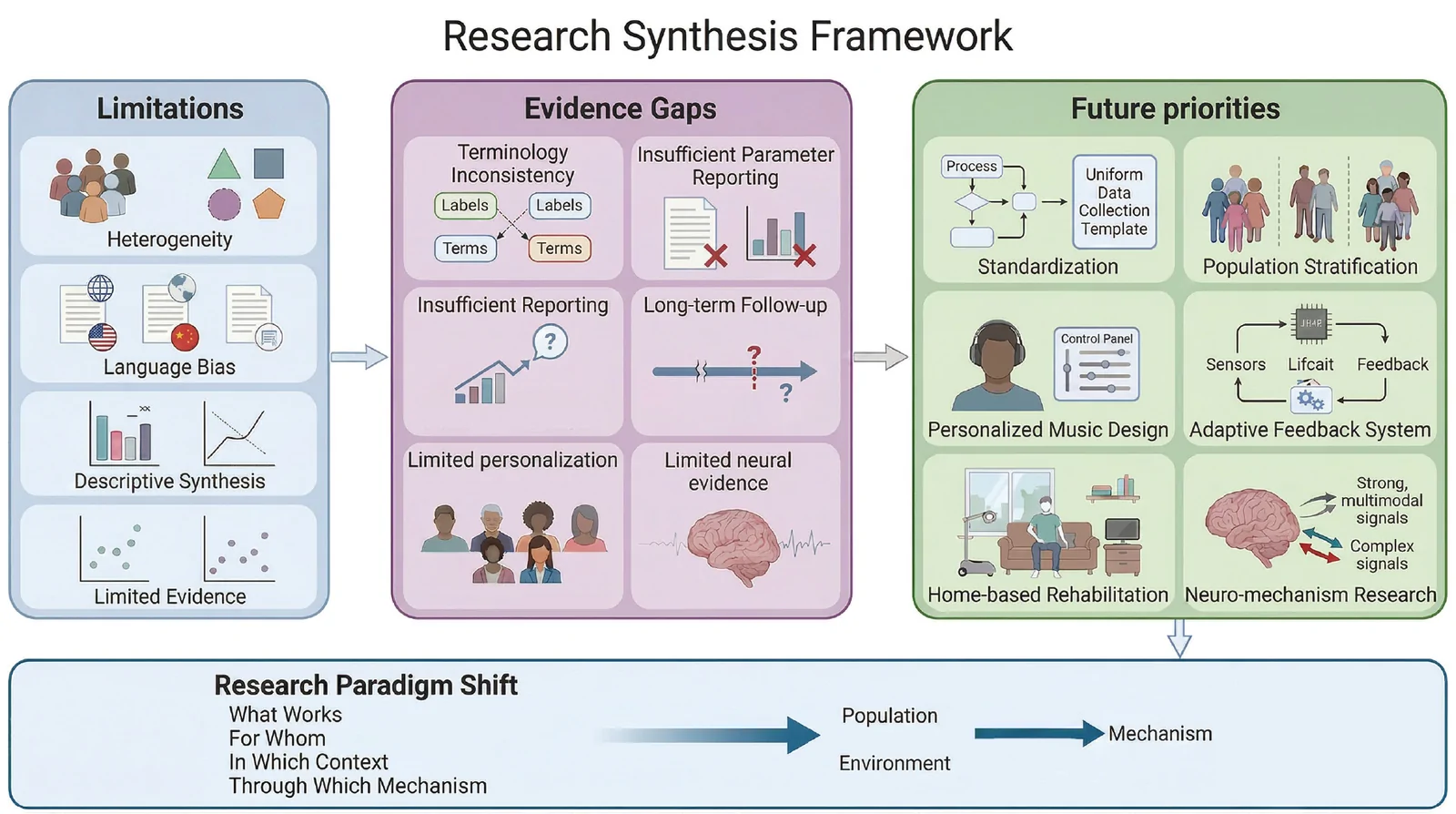
Future research priorities for music-facilitated motor rehabilitation.

### Conclusion

Overall, rhythm-centred and movement-directed music interventions appear promising for supporting motor timing, coordination, and rehabilitation-related task performance, but the current evidence should be interpreted cautiously because the synthesis is descriptive and the included studies vary substantially in population, design, dose, and methodological rigour. Rhythm may play an important role by providing an external temporal structure, promoting sensorimotor coupling, and supporting the temporal organization of movement, whereas other musical elements and interactive feedback may contribute in more task-specific ways.

Based on current evidence, research on music intervention has focused primarily on coordination-related outcomes, lower-limb and gait function, and fine-motor skills. This pattern suggests that the field has largely focused on tasks that require substantial rhythmic synchronization and precise temporal control of movement. By contrast, studies addressing whole-body coordination, complex multi-joint actions, and specific populations have remained relatively limited, indicating considerable room to broaden the scope of research and extend application contexts in the future.

In terms of intervention design, the existing literature has generally adopted a function-oriented approach, with greater emphasis on selecting musical stimuli based on task demands rather than tailoring them to participants’ individual preferences or cultural backgrounds. Although this tendency has, to some extent, improved task specificity, it also highlights the field’s current limitations in individualized design and cultural adaptation. At the same time, the widespread use of technology-assisted assessment has substantially advanced the field towards more objective, fine-grained, and digitalized evaluation. The introduction of sensors, motion capture systems, gait analysis tools, and music-interactive platforms has enabled more detailed quantification of motor performance and functional change, while also providing important support for understanding intervention effects and their underlying mechanisms.

Taken together, the findings of this review indicate that music-facilitated movement does not depend on a single mechanism, but instead emerges from the combined action of multiple musical elements through pathways involving rhythmic synchronization, sensorimotor integration, attentional modulation, and feedback support. Different application contexts and movement types correspond to distinct intervention pathways: rhythm-driven strategies have been used more often in gait and lower-limb rehabilitation, whereas interactive musical engagement has been applied more often in upper-limb and fine-motor training. This pattern suggests that the effectiveness of music intervention depends largely on the degree of alignment among musical features, task demands, and individual characteristics.

Looking ahead, future research should strengthen standardized terminology, full reporting of intervention parameters, transparent screening documentation, population-specific interpretation, and outcome-measure consistency. Individualized music design, adaptive rhythmic cueing, home-based technologies, and multimodal assessment may help improve translational relevance, but these approaches also require attention to feasibility, clinician training, cost, accessibility, and user adherence. Overall, the central task of future research is not only to determine whether music-based or rhythm-centred strategies can support movement, but also to specify for whom, under which task and dosing conditions, through which mechanisms, and with what implementation requirements they may be most useful.

## Supplementary Material




