# Supplementary material for: RHYTHM-CENTRED MUSIC INTERVENTIONS FOR MOTOR COORDINATION AND REHABILITATION: A SCOPING REVIEW OF MECHANISMS, DESIGN PRINCIPLES, AND TECHNOLOGY-ASSISTED ASSESSMENT
Source: J Rehabil Med. 2026 Oct 12;58:46015. doi: 10.2340/jrm.v58.46015 (PMC13670361; doi:10.2340/jrm.v58.46015)
Supplement: Supplementary file 1 [file JRM-58-46015-s1.pdf]

Appendix A. List of included studies.

Author (Year)	Title	Keywords
**(Bood et al., 2013)**	The Power of Auditory-Motor Synchronization in Sports: Enhancing Running Performance by Coupling Cadence with the Right Beats	auditory-motor synchronization, running performance, cadence, acoustic stimuli, motivational music, metronome, sports performance, perceived exertion, heart rate, and running economy.
**(Dalla Bella et al., 2013)**	Why movement is captured by music, but less by speech: role of temporal regularity	Temporal Regularity, Music, Speech, Sensorimotor Synchronization, Beat Isochrony, Meter, Movement
**(Braun Janzen et al., 2014)**	Timing skills and expertise: discrete and continuous timed movements among musicians and athletes	emergent timing, event timing, expertise, training, music, sports
**(Braun Janzen et al., 2014)**	A developmental study of the effect of music training on timed movements	rhythmic movements, timing, development, music training, discrete movements, continuous movements
(Cos et al., 2015)	Balancing out dwelling and moving: optimal sensorimotor synchronization	motor control, optimality, psychophysics, rhythmic movements, synchronization
(Lang et al., 2015)	Lost in the rhythm: Effects of rhythm on subsequent interpersonal coordination	Rhythm; Interpersonal coordination; Motor coupling; Social bonding
(Ross et al., 2016)	Influence of musical groove on postural sway	groove, entrainment, postural sway, rhythm, musical auditory stimulation
**(Dyer et al., 2017)**	Advantages of melodic over rhythmic movement sonification in bimanual motor skill learning	Bimanual coordination, Skill, Retention, Augmented feedback, Movement sonification
**(Elvers and Steffens, 2017)**	The Sound of Success: Investigating Cognitive and Behavioral Effects of Motivational Music in Sports	music, motivation, self-enhancement, self-esteem, sports, risk-taking, motor coordination

**(Moore et al., 2017)**	Diffusion tensor MRI tractography reveals increased fractional anisotropy (FA) in arcuate fasciculus following music-cued motor training	Neuroplasticity, Arcuate fasciculus, Music-cued motor training, White matter, Diffusion tensor MRI, Auditory-motor training
**(Karageorghis et al., 2017)**	Interactive effects of music tempi and intensities on grip strength and subjective affect	activation, arousal potential, circumplex model, precompetition, strength task
**(McPherson et al., 2018)**	Intrinsic Rhythmicity Predicts Synchronization-Continuation Entrainment Performance	Rhythmic entrainment, intrinsic rhythmicity, synchronization-continuation task, spontaneous motor tempo, tempo-dependent performance
**(Rönnqvist et al., 2018)**	Influences of Synchronized Metronome Training on Soccer Players' Timing Ability, Performance Accuracy, and Lower-Limb Kinematics	timing-training, sensorimotor, kinematics, soccer, cognitive-load
**(Slater et al., 2018)**	Got rhythm? Better inhibitory control is linked with more consistent drumming and enhanced neural tracking of the musical beat in adult percussionists and nonpercussionists	inhibitory control, drumming, musical beat, neural tracking, rhythm skills
**(Karageorghis et al., 2019)**	Effects of auditory rhythm on movement accuracy in dance performance	Acoustic stimuli, Movement error, Sequence, Synchronization, Syncopation
**(Koshimori et al., 2019)**	Motor Synchronization to Rhythmic Auditory Stimulation (RAS) Attenuates Dopaminergic Responses in Ventral Striatum in Young Healthy Adults: [11C]-(+)-PHNO PET Study	finger tapping, rhythmic auditory stimulation, D2/3 receptors, dopamine, PET, [11C]-(+)-PHNO, auditory-motor entrainment, basal ganglia
**(Arbinaga et al., 2020)**	Influence of Music on Closed Motor Skills: A Controlled Study with Novice Female Dart-Throwers	tempo; attention; performance; music; synchronization; motor tasks; closed skills; throwers

**(Kim and Yoo, 2020)**	Rhythm-Motor Dual Task Intervention for Fall Prevention in Healthy Older Adults	dual task, fall prevention, healthy elderly, instrument playing, music intervention
**(Schaffert et al., 2020)**	Development and evaluation of a novel music-based therapeutic device for upper extremity movement training: A pre-clinical, single-arm trial	music-based therapy, upper extremity movement training, neurological rehabilitation, SONATA device, motor function, sensory feedback
**(Su and Keller, 2018)**	Your move or mine? Music training and kinematic compatibility modulate synchronization with self- versus other-generated dance movement	motor simulation, music training, kinematic compatibility, dance movement, synchronization
**(Vathagavorakul et al., 2020)**	The effect of experience in movement coordination with music on polyrhythmic production: Comparison between artistic swimmers and water polo players during eggbeater kick performance	polyrhythmic production, movement coordination, artistic swimming, water polo, eggbeater kick, metronome rhythms
**(Vathagavorakul et al., 2020)**	Differences in limb coordination in polyrhythmic production among water polo players, artistic swimmers and drummers	polyrhythm; limb coordination; artistic swimming; tapping
**(Mårup et al., 2022)**	Coordination of voice, hands and feet in rhythm and beat performance	interlimb coordination, rhythm performance, beat perception, bodily hierarchy, musical expertise
**(Nguyen et al., 2022)**	BEAT PERCEPTION AND PRODUCTION IN MUSICIANS AND DANCERS	beat perception, motion capture, music, dance, movement

**(Yu et al., 2022)**	From spontaneous rhythmic engagement to joint drumming: A gradual development of flexible coordination at approximately 24 months of age	joint drumming, spontaneous motor tempo, tempo adjustment, synchronization, entrainment, inhibitory control, action prediction
**(Aluru et al., 2014)**	The effect of auditory constraints on motor performance depends on the stage of recovery post-stroke	bimanual movements, upper extremity, rehabilitation, motor learning/training, electromyography, task specificity, cerebrovascular disorders
**(Benoit et al., 2014)**	Musically cued gait-raining improves both perceptual and motor timing in Parkinson's disease	Parkinson's disease, auditory cueing, timing, motor behavior, perception
**(Friedman et al., 2014)**	Retraining and assessing hand movement after stroke using the MusicGlove: Comparison with conventional hand therapy and isometric grip training	Stroke rehabilitation device, Hand, Music therapy, Video game therapy, Stroke assessment, Outcome measures
**(Van Vugt et al., 2014)**	Music-supported motor training after stroke reveals no superiority of synchronization in group therapy	stroke rehabilitation, music therapy, motor improvement, synchronization, social, shared experience, mood
**(Villeneuve et al., 2014)**	A piano training program to improve manual dexterity and upper extremity function in chronic stroke survivors	cerebrovascular accident, hand, paresis, learning, music, rehabilitation
**(Pau et al., 2016)**	Effects of physical rehabilitation integrated with rhythmic auditory stimulation on spatio-temporal and kinematic parameters of gait in Parkinson's disease	rhythm, sound, ras, Parkinson, gait, kinematics, spatio-temporal parameters, gait analysis
**(Raghavan et al., 2016)**	Music Upper Limb Therapy-Integrated: An Enriched Collaborative Approach for Stroke Rehabilitation	rehabilitation, functional recovery, music therapy, enriched environment, bodily perception, social participation, psycho-social

		adjustment, sense of self
**(Scholz et al., 2016)**	Sonification of Arm Movements in Stroke Rehabilitation - A Novel Approach in Neurologic Music Therapy	sonification, stroke, neurorehabilitation, neuroplasticity, music-supported therapy
**(Young et al., 2016)**	Auditory cueing in Parkinson's patients with freezing of gait. What matters most: Action-relevance or cue-continuity?	Parkinson's, Freezing of gait, Sensory cueing, Imitation, Auditory observation, Audio-motor priming
**(Bella et al., 2017)**	Gait improvement via rhythmic stimulation in Parkinson's disease is linked to rhythmic skills	rhythmic auditory stimulation, Parkinson’s disease, gait improvement, sensorimotor synchronization, rhythmic skills
**(Harrison et al., 2017)**	The feasibility of singing to improve gait in Parkinson disease	Gait, Cueing, Music, Singing, Parkinson's disease
**(B é gel et al., 2018)**	Rhythm Workers: A music-based serious game for training rhythm skills	Beat perception, music, serious game, synchronization, training, rhythm
**(Lee et al., 2018)**	Gait Training with Bilateral Rhythmic Auditory Stimulation in Stroke Patients: A Randomized Controlled Trial	stroke; rhythmic auditory stimulation; symmetry; gait
**(Braunlich et al., 2018)**	Rhythmic auditory cues shape neural network recruitment in Parkinson's disease during repetitive motor behavior	basal ganglia, beat, entrainment, music therapy, synchronization
**(Harrison et al., 2019)**	Mental Singing Reduces Gait Variability More Than Music Listening for Healthy Older Adults and People With Parkinson Disease	aging, internal cues, Parkinson's disease, rhythm, singing
**(Lei et al., 2019)**	Cognitive effects of rhythmic auditory stimulation in Parkinson’s disease: A P300 study	Temporal predictability, Auditory-motor, synchronization, ERP, Neurodegenerative disease

**(Nikmaram et al., 2019)**	Musical Sonification of Arm Movements in Stroke Rehabilitation Yields Limited Benefits	sonification, stroke, neurorehabilitation, neuroplasticity, music-supported therapy, neurologic music therapy, auditory-motor coupling
**(Rose et al., 2019)**	Music and Metronomes Differentially Impact Motor Timing in People with and without Parkinson's Disease: Effects of Slow, Medium, and Fast Tempi on Entrainment and Synchronization Performances in Finger Tapping, Toe Tapping, and Stepping on the Spot Tasks	rhythmic auditory stimulation, Parkinson’s disease, motor timing, entrainment, synchronization, metronome, music
**(Street et al., 2019)**	Upper limb rehabilitation in chronic stroke using neurologic music therapy: Two contrasting case studies to inform on treatment delivery and patient suitability	Stroke; hemiparesis; music therapy; home care; upper limb rehabilitation; TIMP
**(De Luca et al., 2020)**	Do patients with PD benefit from music assisted therapy plus treadmill-based gait training? An exploratory study focused on behavioral outcomes	Parkinson’s disease; music-enhanced treadmill; advanced rehabilitation
**(Naro et al., 2020)**	A Case-Controlled Pilot Study on Rhythmic Auditory Stimulation-Assisted Gait Training and Conventional Physiotherapy in Patients With Parkinson's Disease Submitted to Deep Brain Stimulation	beta oscillations, deep brain stimulation(DBS), idiopathic Parkinson's disease (iPD), rhythmic auditory stimulation (RAS), treadmill gait training
**(Véron‐Delor et al., 2019)**	Musical sonification improves motor control in Parkinson's disease: a proof of concept with handwriting	Parkinson's disease; handwriting; musical sonification; rehabilitation
**(Dogruoz Karatekin and Icagasioglu,**	The effect of therapeutic instrumental music performance method on upper extremity functions in adolescent cerebral palsy	Cerebral palsy, Neurological music therapy, Piano training, Therapeutic instrumental music performance, Upper extremity rehabilitation

**2021)**		
**(Ghai et al., 2021)**	Neurophysiological Changes Induced by Music-Supported Therapy for Recovering Upper Extremity Function after Stroke: A Case Series	neurorehabilitation; motor learning; functional connectivity; auditory-motor coupling
**(Haire et al., 2021)**	Therapeutic Instrumental Music Training and Motor Imagery in Post-Stroke Upper-Extremity Rehabilitation: A Randomized-Controlled Pilot Study	Music; Rehabilitation; Stroke; Upper extremity
**(Park and Kim, 2021)**	Dual-task-based drum playing with rhythmic cueing on motor and attention control in patients with parkinson’s disease: A preliminary randomized study	Parkinson’s disease; dual-task; rhythmic cueing; rehabilitation; drum playing
**(Fan et al., 2022)**	Effects of rhythmic auditory stimulation on upper-limb movements in patients with Parkinson's disease	Acoustic stimulation, Parkinson’s disease, Arm, Movement, Bradykinesia
**(Kantan et al., 2022)**	A Technical Framework for Musical Biofeedback in Stroke Rehabilitation	Balance, biofeedback, gait, interactive sonification, music intervention, neurorehabilitation, stroke
**(Puyjarinet et al., 2022)**	At-Home Training With a Rhythmic Video Game for Improving Orofacial, Manual, and Gait Abilities in Parkinson's Disease: A Pilot Study	Parkinson’s disease, rhythmic training, music-based interventions, mobile technologies, serious video games
**(Collimore et al., 2023)**	Autonomous Control of Music to Retrain Walking After Stroke	digital therapeutic, music, rhythm, walking, symmetry, economy
**(Kim et al., 2023)**	Movement-specific keyboard playing for hand function in adolescents and young adults with acquired brain injury	keyboard playing, rehabilitation, hand function, adolescents, acquired brain injury

**(Mazhari-Jensen et al., 2023)**	Inpatient stroke survivors with low gait functioning benefit from music interventions during cardiorespiratory exercise: A randomized cross-over trial	Stroke; exercise; music; aerobic; inpatient; cycling
**(Raglio et al., 2023)**	Movement Sonification Techniques to Improve Balance in Parkinson's Disease: A Pilot Randomized Controlled Trial	Parkinson’s disease; neurological rehabilitation; gait; postural balance; sonification; music therapy
**(Carrillo et al., 2024)**	Auditory rhythm facilitates perception and action in children at risk for developmental coordination disorder	auditory rhythm, developmental coordination disorder, sensorimotor synchronization, motor coordination, speech perception
**(Goetschalckx et al., 2024)**	Auditory-motor synchronization in developmental coordination disorder: Effects on interlimb coordination during walking and running	auditory-motor synchronization, developmental coordination disorder, interlimb coordination, running, walking
**(Goetschalckx et al., 2024)**	Interlimb Coordination and Auditory—Motor Synchronization in Children with Developmental Coordination Disorder: Examining Antiphase Knee Movements with Auditory Metronomes While Seated	interlimb coordination; developmental coordination disorder; children; auditory-motor synchronization; motor timing
**(Seebacher et al., 2024)**	Actual and Imagined Music-Cued Gait Training in People with Multiple Sclerosis: A Double-Blind Randomized Parallel Multicenter Trial	multiple sclerosis, physiotherapy, music, motor imagery, walking, fatigue
**(Smith et al., 2024)**	Exploring Different Types of Music Cues for Arm Movements in Adults with Parkinson's	auditory cueing, Parkinson’s disease, patterned sensory training

References

Aluru, V., Lu, Y., Leung, A., Verghese, J., & Raghavan, P. (2014). Effect of Auditory Constraints on Motor Performance Depends on Stage of Recovery Post-Stroke. In *Frontiers in Neurology* (Vol. 5). Frontiers Media SA. https://doi.org/10.3389/fneur.2014.00106

Arbinaga, F., Romero-Pérez, N., Torres-Rosado, L., Fernández-Ozcorta, E. J., & Mendoza-Sierra, M. I. (2020). Influence of Music on Closed Motor Skills: A Controlled Study with Novice Female Dart-Throwers. In *International Journal of Environmental Research and Public Health* (Vol. 17, Issue 11, p. 4146). MDPI AG. https://doi.org/10.3390/ijerph17114146

Bella, S. D., Benoit, C.-E., Farrugia, N., Keller, P. E., Obrig, H., Mainka, S., & Kotz, S. A. (2017). Gait improvement via rhythmic stimulation in Parkinson's disease is linked to rhythmic skills. In *Scientific Reports* (Vol. 7, Issue 1). Springer Science and Business Media LLC. https://doi.org/10.1038/srep42005

Benoit, C.-E., Dalla Bella, S., Farrugia, N., Obrig, H., Mainka, S., & Kotz, S. A. (2014). Musically Cued Gait-Training Improves Both Perceptual and Motor Timing in Parkinsonâ€™s Disease. In *Frontiers in Human Neuroscience* (Vol. 8). Frontiers Media SA. https://doi.org/10.3389/fnhum.2014.00494

Bood, R. J., Nijssen, M., van der Kamp, J., & Roerdink, M. (2013). The Power of Auditory-Motor Synchronization in Sports: Enhancing Running Performance by Coupling Cadence with the Right Beats. In *PLoS ONE* (Vol. 8, Issue 8, p. e70758). Public Library of Science (PLoS). https://doi.org/10.1371/journal.pone.0070758

Braun Janzen, T., Thompson, William F., & Ranvaud, R. (2014). A developmental study of the effect of music training on timed movements. In *Frontiers in Human Neuroscience* (Vol. 8). Frontiers Media SA. https://doi.org/10.3389/fnhum.2014.00801

Braun Janzen, T., Thompson, William Forde, Ammirante, P., & Ranvaud, R. (2014). Timing skills and expertise: discrete and continuous timed movements among musicians and athletes. In *Frontiers in Psychology* (Vol. 5). Frontiers Media SA. https://doi.org/10.3389/fpsyg.2014.01482

Braunlich, K., Seger, C. A., Jentink, K. G., Buard, I., Kluger, B. M., & Thaut, M. H. (2018). Rhythmic auditory cues shape neural network recruitment in Parkinson's disease during repetitive motor behavior. In *European Journal of Neuroscience* (Vol. 49, Issue 6, pp. 849–858). Wiley.

https://doi.org/10.1111/ejn.14227

Bégel, V., Seilles, A., & Dalla Bella, S. (2018). Rhythm Workers. In *Music & Science* (Vol. 1). SAGE Publications. https://doi.org/10.1177/2059204318794369

Carrillo, C., Chang, A., Armstrong, H., Cairney, J., McAuley, J. D., & Trainor, L. J. (2024). Auditory rhythm facilitates perception and action in children at risk for developmental coordination disorder. In *Scientific Reports* (Vol. 14, Issue 1). Springer Science and Business Media LLC. https://doi.org/10.1038/s41598-024-62322-6

Collimore, A. N., Roto Cataldo, A. V., Aiello, A. J., Sloutsky, R., Hutchinson, K. J., Harris, B., Ellis, T., & Awad, L. N. (2023). Autonomous Control of Music to Retrain Walking After Stroke. In *Neurorehabilitation and Neural Repair* (Vol. 37, Issue 5, pp. 255–265). SAGE Publications. https://doi.org/10.1177/15459683231174223

Cos, I., Girard, B., & Guigon, E. (2015). Balancing out dwelling and moving: optimal sensorimotor synchronization. In *Journal of Neurophysiology* (Vol. 114, Issue 1, pp. 146–158). American Physiological Society. https://doi.org/10.1152/jn.00175.2015

Dalla Bella, S., Białuńska, A., & Sowiński, J. (2013). Why Movement Is Captured by Music, but Less by Speech: Role of Temporal Regularity. In *PLoS ONE* (Vol. 8, Issue 8, p. e71945). Public Library of Science (PLoS). https://doi.org/10.1371/journal.pone.0071945

De Luca, R., Latella, D., Maggio, M. G., Leonardi, S., Sorbera, C., Di Lorenzo, G., Balletta, T., Cannavò, A., Naro, A., Impellizzeri, F., & Calabrò, R. S. (2020). Do patients with PD benefit from music assisted therapy plus treadmill-based gait training? An exploratory study focused on behavioral outcomes. In *International Journal of Neuroscience* (Vol. 130, Issue 9, pp. 933–940). Informa UK Limited. https://doi.org/10.1080/00207454.2019.1710147

Dogruoz Karatekin, B., & Icagasioglu, A. (2021). The effect of therapeutic instrumental music performance method on upper extremity functions in adolescent cerebral palsy. In *Acta Neurologica Belgica* (Vol. 121, Issue 5, pp. 1179–1189). Springer Science and Business Media LLC. https://doi.org/10.1007/s13760-021-01618-0

Dyer, J. F., Stapleton, P., & Rodger, M. W. M. (2017). Advantages of melodic over rhythmic movement sonification in bimanual motor skill learning. In *Experimental Brain Research* (Vol. 235, Issue 10, pp. 3129–3140). Springer Science and Business Media LLC. https://doi.org/10.1007/s00221-017-5047-8

Elvers, P., & Steffens, J. (2017). The Sound of Success: Investigating Cognitive and Behavioral Effects of Motivational Music in Sports. In *Frontiers in Psychology* (Vol. 8). Frontiers Media SA. https://doi.org/10.3389/fpsyg.2017.02026

Fan, W., Li, J., Wei, W., Xiao, S.-H., Liao, Z.-J., Wang, S.-M., & Fong, K. N. K. (2022). Effects of rhythmic auditory stimulation on upper-limb movements in patients with Parkinson's disease. In *Parkinsonism & Related Disorders* (Vol. 101, pp. 27–30). Elsevier BV. https://doi.org/10.1016/j.parkreldis.2022.06.020

Friedman, N., Chan, V., Reinkensmeyer, A. N., Beroukhim, A., Zambrano, G. J., Bachman, M., & Reinkensmeyer, D. J. (2014). Retraining and assessing hand movement after stroke using the MusicGlove: comparison with conventional hand therapy and isometric grip training. In *Journal of NeuroEngineering and Rehabilitation* (Vol. 11, Issue 1, p. 76). Springer Science and Business Media LLC. https://doi.org/10.1186/1743-0003-11-76

Ghai, S., Maso, F. D., Ogourtsova, T., Porxas, A.-X., Villeneuve, M., Penhune, V., Boudrias, M.-H., Baillet, S., & Lamontagne, A. (2021). Neurophysiological Changes Induced by Music-Supported Therapy for Recovering Upper Extremity Function after Stroke: A Case Series. In *Brain Sciences* (Vol. 11, Issue 5, p. 666). MDPI AG. https://doi.org/10.3390/brainsci11050666

Goetschalckx, M., Feys, P., Rameckers, E., Moens, B., Leman, M., & Moumdjian, L. (2024). Auditory–motor synchronization in developmental coordination disorder: Effects on interlimb coordination during walking and running. In *Annals of the New York Academy of Sciences*. Wiley. https://doi.org/10.1111/nyas.15228

Goetschalckx, M., Moumdjian, L., Rameckers, E., & Feys, P. (2024). Interlimb Coordination and Auditory—Motor Synchronization in Children with Developmental Coordination Disorder: Examining Antiphase Knee Movements with Auditory Metronomes While Seated. In *Children* (Vol. 11, Issue 10, p. 1195). MDPI AG. https://doi.org/10.3390/children11101195

Haire, C. M., Tremblay, L., Vuong, V., Patterson, K. K., Chen, J. L., Burdette, J. H., Schaffert, N., & Thaut, M. H. (2021). Therapeutic Instrumental Music Training and Motor Imagery in Post-Stroke Upper-Extremity Rehabilitation: A Randomized-Controlled Pilot Study. In *Archives of Rehabilitation Research and Clinical Translation* (Vol. 3, Issue 4, p. 100162). Elsevier BV. https://doi.org/10.1016/j.arrct.2021.100162

Harrison, E. C., Horin, A. P., & Earhart, G. M. (2019). Mental Singing Reduces Gait Variability More Than Music Listening for Healthy Older Adults and People With Parkinson Disease. In *Journal of Neurologic Physical Therapy* (Vol. 43, Issue 4, pp. 204–211). Ovid Technologies (Wolters

Kluwer Health). https://doi.org/10.1097/npt.0000000000000288

Harrison, E. C., McNeely, M. E., & Earhart, G. M. (2017). The feasibility of singing to improve gait in Parkinson disease. In *Gait & Posture* (Vol. 53, pp. 224–229). Elsevier BV. https://doi.org/10.1016/j.gaitpost.2017.02.008

Kantan, P., Spaich, E. G., & Dahl, S. (2022). A Technical Framework for Musical Biofeedback in Stroke Rehabilitation. In *IEEE Transactions on Human-Machine Systems* (Vol. 52, Issue 2, pp. 220–231). Institute of Electrical and Electronics Engineers (IEEE). https://doi.org/10.1109/thms.2021.3137013

Karageorghis, C. I., Cheek, P., Simpson, S. D., & Bigliassi, M. (2017). Interactive effects of music tempi and intensities on grip strength and subjective affect. In *Scandinavian Journal of Medicine & Science in Sports* (Vol. 28, Issue 3, pp. 1166–1175). Wiley. https://doi.org/10.1111/sms.12979

Karageorghis, C. I., Lyne, L. P., Bigliassi, M., & Vuust, P. (2019). Effects of auditory rhythm on movement accuracy in dance performance. In *Human Movement Science* (Vol. 67, p. 102511). Elsevier BV. https://doi.org/10.1016/j.humov.2019.102511

Kim, S. J., & Yoo, G. E. (2020). Rhythm-Motor Dual Task Intervention for Fall Prevention in Healthy Older Adults. In *Frontiers in Psychology* (Vol. 10). Frontiers Media SA. https://doi.org/10.3389/fpsyg.2019.03027

Kim, S. J., Shin, Y.-K., Jeong, E., & Cho, S.-R. (2023). Movement-specific keyboard playing for hand function in adolescents and young adults with acquired brain injury. In *Frontiers in Neurology* (Vol. 13). Frontiers Media SA. https://doi.org/10.3389/fneur.2022.1062615

Koshimori, Y., Strafella, A. P., Valli, M., Sharma, V., Cho, S., Houle, S., & Thaut, M. H. (2019). Motor Synchronization to Rhythmic Auditory Stimulation (RAS) Attenuates Dopaminergic Responses in Ventral Striatum in Young Healthy Adults: [11C]-(+)-PHNO PET Study. In *Frontiers in Neuroscience* (Vol. 13). Frontiers Media SA. https://doi.org/10.3389/fnins.2019.00106

Lang, M., Shaw, D. J., Reddish, P., Wallot, S., Mitkidis, P., & Xygalatas, D. (2015). Lost in the Rhythm: Effects of Rhythm on Subsequent Interpersonal Coordination. In *Cognitive Science* (Vol. 40, Issue 7, pp. 1797–1815). Wiley. https://doi.org/10.1111/cogs.12302

Lee, S., Lee, K., & Song, C. (2018). Gait Training with Bilateral Rhythmic Auditory Stimulation in Stroke Patients: A Randomized Controlled Trial. In *Brain Sciences* (Vol. 8, Issue 9, p. 164). MDPI AG. https://doi.org/10.3390/brainsci8090164

Lei, J., Conradi, N., Abel, C., Frisch, S., Brodski-Guerniero, A., Hildner, M., Kell, C. A., Kaiser, J., & Schmidt-Kassow, M. (2019). Cognitive effects

of rhythmic auditory stimulation in Parkinson's disease: A P300 study. In *Brain Research* (Vol. 1716, pp. 70–79). Elsevier BV. https://doi.org/10.1016/j.brainres.2018.05.016

Mazhari-Jensen, D. S., Jacobsen, S. L., & Jespersen, K. V. (2023). Inpatient stroke survivors with low gait functioning benefit from music interventions during cardiorespiratory exercise: A randomized cross-over trial. In *Nordic Journal of Music Therapy* (Vol. 32, Issue 5, pp. 462–481). Informa UK Limited. https://doi.org/10.1080/08098131.2023.2190403

McPherson, T., Berger, D., Alagapan, S., & Fröhlich, F. (2018). Intrinsic Rhythmicity Predicts Synchronization-Continuation Entrainment Performance. In *Scientific Reports* (Vol. 8, Issue 1). Springer Science and Business Media LLC. https://doi.org/10.1038/s41598-018-29267-z

Moore, E., Schaefer, R. S., Bastin, M. E., Roberts, N., & Overy, K. (2017). Diffusion tensor MRI tractography reveals increased fractional anisotropy (FA) in arcuate fasciculus following music-cued motor training. In *Brain and Cognition* (Vol. 116, pp. 40–46). Elsevier BV. https://doi.org/10.1016/j.bandc.2017.05.001

Mårup, S. H., Møller, C., & Vuust, P. (2022). Coordination of voice, hands and feet in rhythm and beat performance. In *Scientific Reports* (Vol. 12, Issue 1). Springer Science and Business Media LLC. https://doi.org/10.1038/s41598-022-11783-8

Naro, A., Pignolo, L., Sorbera, C., Latella, D., Billeri, L., Manuli, A., Portaro, S., Bruschetta, D., & Calabrò, R. S. (2020). A Case-Controlled Pilot Study on Rhythmic Auditory Stimulation-Assisted Gait Training and Conventional Physiotherapy in Patients With Parkinson's Disease Submitted to Deep Brain Stimulation. In *Frontiers in Neurology* (Vol. 11). Frontiers Media SA. https://doi.org/10.3389/fneur.2020.00794

Nguyen, T., Sidhu, R. K., Everling, J. C., Wickett, M. C., Gibbings, A., & Grahn, J. A. (2022). Beat Perception and Production in Musicians and Dancers. In *Music Perception: An Interdisciplinary Journal* (Vol. 39, Issue 3, pp. 229–248). University of California Press. https://doi.org/10.1525/mp.2022.39.3.229

Nikmaram, N., Scholz, D. S., Großbach, M., Schmidt, S. B., Spogis, J., Belardinelli, P., Müller-Dahlhaus, F., Remy, J., Ziemann, U., Rollnik, J. D., & Altenmüller, E. (2019). Musical Sonification of Arm Movements in Stroke Rehabilitation Yields Limited Benefits. In *Frontiers in Neuroscience* (Vol. 13). Frontiers Media SA. https://doi.org/10.3389/fnins.2019.01378

Park, J.-K., & Kim, S. J. (2021). Dual-Task-Based Drum Playing with Rhythmic Cueing on Motor and Attention Control in Patients with Parkinson's Disease: A Preliminary Randomized Study. In *International Journal of Environmental Research and Public Health* (Vol. 18, Issue 19, p. 10095).

MDPI AG. https://doi.org/10.3390/ijerph181910095

Pau, M., Corona, F., Pili, R., Casula, C., Sors, F., Agostini, T., Cossu, G., Guicciardi, M., & Murgia, M. (2016). Effects of Physical Rehabilitation Integrated with Rhythmic Auditory Stimulation on Spatio-Temporal and Kinematic Parameters of Gait in Parkinson's Disease. In *Frontiers in Neurology* (Vol. 7). Frontiers Media SA. https://doi.org/10.3389/fneur.2016.00126

Puyjarinet, F., Bégel, V., Geny, C., Driss, V., Cuartero, M.-C., De Cock, V. C., Pinto, S., & Dalla Bella, S. (2022). At-Home Training With a Rhythmic Video Game for Improving Orofacial, Manual, and Gait Abilities in Parkinson's Disease: A Pilot Study. In *Frontiers in Neuroscience* (Vol. 16). Frontiers Media SA. https://doi.org/10.3389/fnins.2022.874032

Raghavan, P., Geller, D., Guerrero, N., Aluru, V., Eimicke, J. P., Teresi, J. A., Ogedegbe, G., Palumbo, A., & Turry, A. (2016). Music Upper Limb Therapy—Integrated: An Enriched Collaborative Approach for Stroke Rehabilitation. In *Frontiers in Human Neuroscience* (Vol. 10). Frontiers Media SA. https://doi.org/10.3389/fnhum.2016.00498

Raglio, A., De Maria, B., Parati, M., Giglietti, A., Premoli, S., Salvaderi, S., Molteni, D., Ferrante, S., & Dalla Vecchia, L. A. (2023). Movement Sonification Techniques to Improve Balance in Parkinson's Disease: A Pilot Randomized Controlled Trial. In *Brain Sciences* (Vol. 13, Issue 11, p. 1586). MDPI AG. https://doi.org/10.3390/brainsci13111586

Rose, D., Delevoye-Turrell, Y., Ott, L., Annett, L. E., & Lovatt, P. J. (2019). Music and Metronomes Differentially Impact Motor Timing in People with and without Parkinson's Disease: Effects of Slow, Medium, and Fast Tempi on Entrainment and Synchronization Performances in Finger Tapping, Toe Tapping, and Stepping on the Spot Tasks. In *Parkinson's Disease* (Vol. 2019, pp. 1–18). Wiley. https://doi.org/10.1155/2019/6530838

Ross, J. M., Warlaumont, A. S., Abney, D. H., Rigoli, L. M., & Balasubramaniam, R. (2016). Influence of musical groove on postural sway. In *Journal of Experimental Psychology: Human Perception and Performance* (Vol. 42, Issue 3, pp. 308–319). American Psychological Association (APA). https://doi.org/10.1037/xhp0000198

Rönnqvist, L., McDonald, R., & Sommer, M. (2018). Influences of Synchronized Metronome Training on Soccer Players' Timing Ability, Performance Accuracy, and Lower-Limb Kinematics. In *Frontiers in Psychology* (Vol. 9). Frontiers Media SA. https://doi.org/10.3389/fpsyg.2018.02469

Schaffert, N., Braun Janzen, T., Ploigt, R., Schlüter, S., Vuong, V., & Thaut, M. H. (2020). Development and evaluation of a novel music-based therapeutic device for upper extremity movement training: A pre-clinical, single-arm trial. In *PLOS ONE* (Vol. 15, Issue 11, p. e0242552). Public Library of Science (PLoS). https://doi.org/10.1371/journal.pone.0242552

Scholz, D. S., Rohde, S., Nikmaram, N., Brückner, H.-P., Großbach, M., Rollnik, J. D., & Altenmüller, E. O. (2016). Sonification of Arm Movements in Stroke Rehabilitation – A Novel Approach in Neurologic Music Therapy. In *Frontiers in Neurology* (Vol. 7). Frontiers Media SA. https://doi.org/10.3389/fneur.2016.00106

Seebacher, B., Helmlinger, B., Pinter, D., Heschl, B., Ehling, R., Hechenberger, S., Reindl, M., Khalil, M., Enzinger, C., Deisenhammer, F., & Brenneis MD, C. (2024). Actual and Imagined Music-Cued Gait Training in People with Multiple Sclerosis: A Double-Blind Randomized Parallel Multicenter Trial. In *Neurorehabilitation and Neural Repair* (Vol. 38, Issue 8, pp. 555–569). SAGE Publications. https://doi.org/10.1177/15459683241260724

Slater, J., Ashley, R., Tierney, A., & Kraus, N. (2018). Got Rhythm? Better Inhibitory Control Is Linked with More Consistent Drumming and Enhanced Neural Tracking of the Musical Beat in Adult Percussionists and Nonpercussionists. In *Journal of Cognitive Neuroscience* (Vol. 30, Issue 1, pp. 14–24). MIT Press - Journals. https://doi.org/10.1162/jocn_a_01189

Smith, R. A. , Naomi, D. , & Blythe, L. A. . (2024). Exploring different types of music cues for arm movements in adults with parkinson's. #i{Music Therapy Perspectives}(2), 2.

Street, A. J., Fachner, J., & Magee, W. L. (2019). Upper limb rehabilitation in chronic stroke using neurologic music therapy: Two contrasting case studies to inform on treatment delivery and patient suitability. In *Nordic Journal of Music Therapy* (Vol. 28, Issue 5, pp. 382–404). Informa UK Limited. https://doi.org/10.1080/08098131.2019.1606848

Su, Y.-H., & Keller, P. E. (2018). Your move or mine? Music training and kinematic compatibility modulate synchronization with self- versus other-generated dance movement. In *Psychological Research* (Vol. 84, Issue 1, pp. 62–80). Springer Science and Business Media LLC. https://doi.org/10.1007/s00426-018-0987-6

Van Vugt, F. T., Ritter, J., Rollnik, J. D., & Altenmüller, E. (2014). Music-supported motor training after stroke reveals no superiority of synchronization in group therapy. In *Frontiers in Human Neuroscience* (Vol. 8). Frontiers Media SA. https://doi.org/10.3389/fnhum.2014.00315

Vathagavorakul, R., Gonjo, T., & Homma, M. (2020a). Differences in limb coordination in polyrhythmic production among water polo players, artistic swimmers and drummers. In *Journal of Motor Behavior* (Vol. 53, Issue 2, pp. 191–199). Informa UK Limited. https://doi.org/10.1080/00222895.2020.1748860

Vathagavorakul, R., Gonjo, T., & Homma, M. (2020b). The effect of experience in movement coordination with music on polyrhythmic production: Comparison between artistic swimmers and water polo players during eggbeater kick performance. In *PLOS ONE* (Vol. 15, Issue 8, p. e0238197). Public Library of Science (PLoS). https://doi.org/10.1371/journal.pone.0238197

Villeneuve, M., Penhune, V., & Lamontagne, A. (2014). A Piano Training Program to Improve Manual Dexterity and Upper Extremity Function in Chronic Stroke Survivors. In *Frontiers in Human Neuroscience* (Vol. 8). Frontiers Media SA. https://doi.org/10.3389/fnhum.2014.00662

Véron-Delor, L., Pinto, S., Eusebio, A., Azulay, J., Witjas, T., Velay, J., & Danna, J. (2019). Musical sonification improves motor control in Parkinson's disease: a proof of concept with handwriting. In *Annals of the New York Academy of Sciences* (Vol. 1465, Issue 1, pp. 132–145). Wiley. https://doi.org/10.1111/nyas.14252

Young, W. R., Shreve, L., Quinn, E. J., Craig, C., & Bronte-Stewart, H. (2016). Auditory cueing in Parkinson's patients with freezing of gait. What matters most: Action-relevance or cue-continuity? In *Neuropsychologia* (Vol. 87, pp. 54–62). Elsevier BV. https://doi.org/10.1016/j.neuropsychologia.2016.04.034

Yu, L., Todoriki, K., & Myowa, M. (2022). From spontaneous rhythmic engagement to joint drumming: A gradual development of flexible coordination at approximately 24 months of age. In *Frontiers in Psychology* (Vol. 13). Frontiers Media SA. https://doi.org/10.3389/fpsyg.2022.907834
