# Supplementary material for: RHYTHM-CENTRED MUSIC INTERVENTIONS FOR MOTOR COORDINATION AND REHABILITATION: A SCOPING REVIEW OF MECHANISMS, DESIGN PRINCIPLES, AND TECHNOLOGY-ASSISTED ASSESSMENT
Source: J Rehabil Med. 2026 Oct 12;58:46015. doi: 10.2340/jrm.v58.46015 (PMC13670361; doi:10.2340/jrm.v58.46015)
Supplement: Supplementary file 2 [file JRM-58-46015-s2.pdf]

Table SI. Detailed classification of technology-assisted and related assessment tools

Main category	Subcategory	Representative tools/systems
Technology-assisted assessments	Inertial sensors and wearable devices	Physilog 5, Xsens, Seeeduino XIAO, IMU, ActiGraph GT3X, wireless IMU systems
	Motion capture systems	Vicon MX, OptiTrack, VENUS3D, ProReflex, Qualisys, CODA, Optotrak, Smart-D
	Tablets and touch devices	LG G Pad 8.0, Apple single-button mouse, mouse pad, Wacom Intuos3 tablet
	Audio and tapping data recording tools	Audacity, modified Peregrine glove, vibration sensor/LabJack, drum piezo sensor/DAQ, SPD ONE Percussion, Acoustim8/BioPac
	Music interaction systems and software	Cubase, DGX-230 MIDI keyboard, Synthesia, GarageBand, Thumbjam, MusicGlove, IsoTrainer, Rhythm Workers game
	Gait analysis systems	GAITRite Walkway, Gait Trainer 3
	Video synchronization systems	The Motion Monitor
Objective standardized measures	Upper-limb and fine motor scales	Nine-Hole Peg Test, Box and Block Test, Jebsen Hand Function Test, Purdue Pegboard Test, ARAT
	Motor function and rehabilitation scales	Fugl-Meyer Assessment, Wolf Motor Function Test-FAS, Manual Ability Classification System
	Gait, balance, and functional tests	Timed Up and Go Test, Berg Balance Scale
	Coordination tests	Finger-to-nose test, Finger-tapping test
	Strength measures	Hydraulic Hand Dynamometer, Hydraulic Pinch Gauge

Main category	Subcategory	Representative tools/systems
	Accuracy and task-performance measures	Ball throwing task, task completion time, step count
	Rhythm and auditory measures	Beat Alignment Test, BAASTA battery
Neurophysiological indicators	Neural and physiological measures	EEG, MEG, ECG, EMG, motor-cognitive ERP, CoP
Subjective assessments	Observer-based ratings	Observer ratings of rhythm-gait synchrony
	Subjective improvement ratings	Subjective ratings of motor improvement or synchrony

Note: Tool names were grouped according to the categories reported in the original studies. Individual studies may have used more than one tool or more than one type of assessment.

Table SII. Database-specific search strategies

Database	Search field/syntax	Search string
PubMed	Title/Abstract; human studies; 2013/01/01-2024/12/31	(music OR rhythm* OR "rhythmic auditory stimulation" OR "rhythmic auditory cueing" OR melody OR "auditory stimulation") AND ("motor coordination" OR "motor control" OR "movement quality" OR gait OR balance OR "upper limb" OR "fine motor" OR "movement performance") AND (rehabilitation OR "motor training" OR exercise OR "functional outcome" OR "task performance")
Scopus	TITLE-ABS-KEY; English articles; 2013-2024	TITLE-ABS-KEY((music OR rhythm* OR "rhythmic auditory stimulation" OR "rhythmic auditory cueing" OR melody OR "auditory stimulation") AND ("motor coordination" OR "motor control" OR "movement quality" OR gait OR balance OR "upper limb" OR "fine motor" OR "movement performance") AND (rehabilitation OR "motor training" OR exercise OR "functional outcome" OR "task performance"))
Web of Science	Topic search; English articles; 2013-2024	TS=((music OR rhythm* OR "rhythmic auditory stimulation" OR "rhythmic auditory cueing" OR melody OR "auditory stimulation") AND ("motor coordination" OR "motor control" OR "movement quality" OR gait OR balance OR "upper limb" OR "fine motor" OR "movement performance") AND (rehabilitation OR "motor training" OR exercise OR "functional outcome" OR "task performance"))

Database	Search field/syntax	Search string
PsycINFO	Title/Abstract/Subject; peer-reviewed; English; 2013-2024	((music OR rhythm* OR "rhythmic auditory stimulation" OR "rhythmic auditory cueing" OR melody OR "auditory stimulation") AND ("motor coordination" OR "motor control" OR "movement quality" OR gait OR balance OR "upper limb" OR "fine motor" OR "movement performance") AND (rehabilitation OR "motor training" OR exercise OR "functional outcome" OR "task performance"))

Table SIII. Study selection and screening transparency

Stage	Number	Details
Records identified from databases	1,540	PubMed 8; Scopus 941; Web of Science 463; PsycINFO 128
Records identified from other sources	0	Backward and supplementary source checking did not add eligible records
Records after duplicates removed	1,181	Duplicates removed before screening
Records excluded at title/abstract screening	1,059	Clearly irrelevant to movement-directed music/rhythm intervention
Full-text reports assessed	122	Full texts screened against PCC criteria
Full-text reports excluded	59	Not music-based intervention or music not used as the primary movement-directed medium
Studies included	63	Included in descriptive scoping synthesis
Reviewer process	Two reviewers	Independent screening followed by consensus discussion and third-reviewer consultation when needed

Table SIV. Frequency summary supporting cross-domain comparisons

Variable	Category	Frequency	Interpretation for cross-domain synthesis
Core musical element	Rhythm	46/63 (73%)	Most frequent element; relevant to temporal cueing and synchronization
Core musical element	Melody	13/63 (20%)	Supportive, sequential, or motivational role
Core musical element	Integrated elements	3/63 (5%)	Combined musical strategies remained uncommon
Core musical element	Pitch	1/63 (2%)	Rarely the primary movement-guiding element
Delivery mode	Melody listening	25/63 (40%)	Common listening-based format
Delivery mode	Rhythm listening/rhythmic cueing	20/63 (32%)	Common in gait and timing-oriented tasks
Delivery mode	Instrument playing	11/63 (17%)	Relevant to upper-limb and fine-motor engagement
Delivery mode	Singing	1/63 (2%)	Rare as a primary intervention format
Delivery mode	Hybrid interventions	6/63 (9%)	Combined cueing, feedback, and rehabilitation formats
Target movement	Lower limb/gait	26 studies	Often paired with rhythmic cueing and gait or wearable assessment
Target movement	Fine motor skills	26 studies	Often paired with active, tablet-based, or interactive music tasks
Target movement	Upper limb	18 studies	Linked to dexterity, accuracy, and hand-function tests
Target movement	Body coordination	5 studies	Underrepresented area
Assessment	Technology-assisted	52 occurrences	Dominant measurement approach

Variable	Category	Frequency	Interpretation for cross-domain synthesis
	assessments		
Assessment	Objective standardized measures	18 occurrences	Structured functional outcomes
Assessment	Neurophysiological indicators	6 occurrences	Limited but important mechanistic evidence
Assessment	Subjective assessments	2 occurrences	Least common category
